# Effects of post-acute COVID-19 syndrome on the functional brain networks of non-hospitalized individuals

**DOI:** 10.3389/fneur.2023.1136408

**Published:** 2023-03-27

**Authors:** Nathan W. Churchill, Eugenie Roudaia, J. Jean Chen, Asaf Gilboa, Allison Sekuler, Xiang Ji, Fuqiang Gao, Zhongmin Lin, Aravinthan Jegatheesan, Mario Masellis, Maged Goubran, Jennifer S. Rabin, Benjamin Lam, Ivy Cheng, Robert Fowler, Chris Heyn, Sandra E. Black, Bradley J. MacIntosh, Simon J. Graham, Tom A. Schweizer

**Affiliations:** ^1^Neuroscience Research Program, St. Michael’s Hospital, Toronto, ON, Canada; ^2^Keenan Research Centre for Biomedical Science of St. Michael’s Hospital, Toronto, ON, Canada; ^3^Physics Department, Toronto Metropolitan University, Toronto, ON, Canada; ^4^Rotman Research Institute, Baycrest Academy for Research and Education, Toronto, ON, Canada; ^5^Department of Medical Biophysics, University of Toronto, Toronto, ON, Canada; ^6^Institute of Biomedical Engineering, University of Toronto, Toronto, ON, Canada; ^7^Department of Psychology, University of Toronto, Toronto, ON, Canada; ^8^Department of Psychology, Neuroscience and Behaviour, McMaster University, Hamilton, ON, Canada; ^9^LC Campbell Cognitive Neurology Research Group, Sunnybrook Health Sciences Centre, Toronto, ON, Canada; ^10^Hurvitz Brain Sciences Program, Sunnybrook Research Institute, Toronto, ON, Canada; ^11^Physical Sciences Platform, Sunnybrook Research Institute, Toronto, ON, Canada; ^12^Division of Neurology, Department of Medicine, Sunnybrook Health Sciences Centre, University of Toronto, Toronto, ON, Canada; ^13^Harquail Centre for Neuromodulation, Sunnybrook Research Institute, Toronto, ON, Canada; ^14^Rehabilitation Sciences Institute, University of Toronto, Toronto, ON, Canada; ^15^Evaluative Clinical Sciences, Sunnybrook Research Institute, Toronto, ON, Canada; ^16^Integrated Community Program, Sunnybrook Research Institute, Toronto, ON, Canada; ^17^Department of Medicine, University of Toronto, Toronto, ON, Canada; ^18^Emergency and Critical Care Research Program, Sunnybrook Research Institute, Toronto, ON, Canada; ^19^Department of Medical Imaging, University of Toronto, Toronto, ON, Canada; ^20^Computational Radiology and Artificial Intelligence Unit, Division of Radiology and Nuclear Medicine, Oslo University Hospital, Oslo, Norway; ^21^Faculty of Medicine (Neurosurgery), University of Toronto, Toronto, ON, Canada

**Keywords:** brain function, COVID-19, functional connectivity, symptoms, fMRI

## Abstract

**Introduction:**

The long-term impact of COVID-19 on brain function remains poorly understood, despite growing concern surrounding post-acute COVID-19 syndrome (PACS). The goal of this cross-sectional, observational study was to determine whether there are significant alterations in resting brain function among non-hospitalized individuals with PACS, compared to symptomatic individuals with non-COVID infection.

**Methods:**

Data were collected for 51 individuals who tested positive for COVID-19 (mean age 41±12 yrs., 34 female) and 15 controls who had cold and flu-like symptoms but tested negative for COVID-19 (mean age 41±14 yrs., 9 female), with both groups assessed an average of 4-5 months after COVID testing. None of the participants had prior neurologic, psychiatric, or cardiovascular illness. Resting brain function was assessed *via* functional magnetic resonance imaging (fMRI), and self-reported symptoms were recorded.

**Results:**

Individuals with COVID-19 had lower temporal and subcortical functional connectivity relative to controls. A greater number of ongoing post-COVID symptoms was also associated with altered functional connectivity between temporal, parietal, occipital and subcortical regions.

**Discussion:**

These results provide preliminary evidence that patterns of functional connectivity distinguish PACS from non-COVID infection and correlate with the severity of clinical outcome, providing novel insights into this highly prevalent disorder.

## 1. Introduction

Severe acute respiratory syndrome coronavirus-2 (SARS-CoV-2) and the associated coronavirus disease 2019 (COVID-19) (1) represent an unprecedented global health crisis. In addition to its effects on the respiratory system, COVID-19 can significantly impact multiple organ systems, including the brain. Acute effects on the central nervous system are well documented, with hyposmia, hypogeusia, headaches and cognitive disturbances being widely reported (2). There is also evidence of neuropathology, with punctate lesions observed on clinical imaging of individuals with acute COVID-19 (3, 4), and signs of neuroinflammation, hypoxia, microvascular injury and axonal degeneration in the autopsied brains of individuals who died while infected with SARS-CoV-2 (5–8). Similar findings have also been reported in non-human primate studies, even in the absence of severe respiratory disease (9–11).

Collectively, these findings raise concerns about the long-term effects of COVID-19 on brain function in humans. Such concerns are further bolstered by growing case numbers of post-acute COVID-19 syndrome (PACS), in which symptoms and neurological issues associated with SARS-CoV-2 infection persist for more than 12 weeks post-infection (12, 13), i.e., well outside of the acute phase of the disease. This disorder has a relatively high prevalence, with conservative estimates of at least 30% among COVID-19 survivors (14). At present, however, we have a limited understanding of the functional brain changes that are associated with PACS, especially among non-hospitalized cohorts. In particular, it is unclear to what extent functional changes differ from non-COVID viral infection, and whether these changes are correlated with the severity of symptom burden. This limits our ability to identify at-risk individuals and to develop therapeutic interventions.

Blood-oxygenation-level dependent functional MRI (BOLD fMRI) provides the means to investigate the effects of PACS on brain function at the network level, based on fluctuations in local blood-oxygen levels. This technique has been used to study brain function in numerous clinical cohorts, typically by estimating functional connectivity, which reflects the temporal synchronization between brain regions. The present cross-sectional observational study examined resting-state BOLD fMRI data collected as part of the Toronto-based NeuroCOVID-19 study (15). It compared whole-brain functional networks of self-isolating individuals who tested positive for SARS-CoV-2 and subsequently experienced persistent symptoms, relative to controls who tested negative but had cold or flulike symptoms, with both groups imaged an average of 4–5 months after COVID testing. There is emerging evidence of long-term declines in cerebral blood flow (CBF) (16) and grey matter (17, 18) among non-hospitalized individuals with persistent symptoms, as well as neurometabolic deficits throughout the recovery timeline (19, 20), particularly in frontal, temporal and subcortical regions. Thus, we hypothesized that the COVID-19 group would have decreased network connectivity in these regions relative to controls, measured using BOLD fMRI. As the determination of PACS is largely based on symptom presentation, subsequent analyzes of the COVID-19 cohort also examined the relationship between self-reported symptoms and functional connectivity.

## 2. Materials and methods

### 2.1. Study participants

Participants with COVID-19 were recruited through the Department of Emergency Medicine at Sunnybrook Health Sciences Centre, Toronto, Canada; physician referral; and community advertisements, following a diagnosis of COVID-19. Diagnosis was determined in accordance with local provincial public health procedures (21), and included a nasopharyngeal or oropharyngeal swab with real-time reverse transcription polymerase chain reaction (PCR) testing, conducted at a provincially-approved facility. Participants were assessed at a minimum of 14 days post-infection and did not travel in this period. In addition, the study recruited controls who had symptoms of viral illness but tested negative for COVID-19. None of the participants had a history of neurologic or psychiatric illness, unstable cardiovascular disease, or MRI contraindications. Recruitment and data collection were carried out between May 2020 and December 2021, and the study was in accordance with the Canadian Tri-Council Policy Statement 2, with full approval of the study by the Sunnybrook Health Sciences Centre ethics board and with participants giving free and written informed consent.

### 2.2. Magnetic resonance imaging data

All participants were imaged at Sunnybrook Health Sciences Centre using a 3 Tesla Magnetom Prisma MRI system (Siemens Healthineers). Structural and functional imaging included a T1-weighted 3-dimensional magnetization-prepared rapid gradient-echo (MPRAGE) anatomical scan (sagittal acquisition, 1.0 mm isotropic voxels) and a 2-dimensional multi-slice blood-oxygenation-level–dependent (BOLD) resting state functional MRI scan (3.5 mm isotropic voxels, 30/2130 ms TE/TR, 250 volumes). The data were processed using a hybrid pipeline that included ANTs (advanced normalization tools; http://stnava.github.io/ANTs), AFNI (analysis of functional neuroimages; https://afni.nimh.nih.gov), FSL (FMRIB software library; https://fsl.fmrib.ox.ac.uk/fsl) and custom in-house software (see Appendix 1 for details of acquisition and preprocessing), with the final data in Montreal Neurological Institute (MNI) coordinate space and voxels resampled at 3mm isotropic resolution. Afterwards, the brain was parcellated based on the Brainnetome atlas (BNA) with cohort-specific weights obtained *via* mixture model fitting (22). This approach subdivided the brain into 246 regions of interest (ROIs), with 2 ROIs in bilateral inferior temporal gyri discarded due to susceptibility-related signal dropout. Pairwise functional connectivity was then computed between all remaining nodes, producing a 244×244 matrix with 29,646 unique connectivity values per participant. Outlier imaging data were identified in terms of both estimated head motion and BOLD signal fluctuations, with two participants (controls) excluded from imaging analysis; further post-hoc testing of head motion found no significant confounding effects on the main study analyses (see Appendix 1 for details).

### 2.3. Analysis of clinical and demographic data

Participant demographics are listed in Table 1, including age, sex and years of education; the time interval from symptom onset to imaging, and from PCR test to imaging were also reported. All participants completed a questionnaire evaluating symptom status for 9 items: fever, cough, sore throat, shortness of breath, fatigue, gastrointestinal issues, problems with smell/taste, headache and “other.” They reported whether each symptom (1) was absent, (2) had occurred but resolved, or (3) was currently ongoing. Along with individual symptom ratings, overall severity scores were obtained by summing the number of “ongoing” and “resolved” symptoms, and the “combined” total of both ongoing and resolved symptoms. Symptom counts are an established approach for measuring the severity of clinical outcome in multiple cohorts, including concussion, mild behavioral impairment and mild cognitive impairment (23–25). The approximate normality of each demographic variable was assessed by comparing its skewness and kurtosis against simulated null distributions, i.e., with the statistics calculated from normally-distributed samples (5,000 iterations). The means and standard deviations were reported for measures that did not deviate from normality at *p* < 0.05 and the medians with upper and lower quartiles were reported for those that did. The frequency of ongoing and resolved individual symptoms was also reported, with bootstrapped 95% confidence intervals (95%CIs) obtained by resampling on participants with replacement (2000 iterations).

**Table 1:** Summary of demographic and clinical data for study participants.

	Controls (*N* = 15)	COVID-19 (*N* = 51)	Group diff (SE)	Statistics of effect	
				BSR	*p*-value
Age, mean (SD)	41.4 (14.0) yrs.	41.3 (11.9) yrs.	−0.3 (3.8) yrs.	−0.02	0.980
Female, total (percent)	9/15 (60.0%)	34/51 (66.7%)	6.7 (14.1) %	0.42	0.635
Education, mean (SD)	16.4 (2.8) yrs.	16.3 (2.2) yrs.	−0.1 (0.8) yrs.	−0.10	0.912
Days (onset to scan)	180 [138, 218]	117 [83, 185]	−63 (29)	−2.18	0.063
Days (test to scan)	136 [55, 195]	113 [70, 177]	−23 (33)	−0.68	0.496
Symptom count (ongoing)	1.9 (2.4)	2.1 (2.3)	0.2 (0.7)	0.30	0.744
Symptom count (resolved)	2.9 (2.3)	4.7 (1.8)	1.8 (0.6)	2.96	0.003
Symptom count (combined)	4.8 (3.6)	6.8 (2.6)	2.0 (0.9)	2.15	0.031

For group distributions, the mean and standard deviation (SD) are reported for normally-distributed data, while the median is reported with lower and upper distribution quartiles [Q1, Q3] for non-normal data. For group comparisons, the mean difference and bootstrapped standard error (SE) are reported, along with the bootstrap ratio (BSR) and percentile-based *p*-value.

A series of 2-sample bootstrap analyzes then tested for group differences in demographics (2-tailed) and in the frequencies of individual symptoms, along with the total number of ongoing, resolved and combined symptoms. Reporting included mean differences, standard errors (SE), bootstrap ratios (BSR; a z-scored statistic of effect, based on the ratio of bootstrap mean/SE) and percentile *p*-values (2-tailed). For analyzes of both clinical and neuroimaging data, bootstrapping was used to estimate effects, as this non-parametric approach is robust to deviations from normality and unequal variances, particularly given the unbalanced group sample sizes (26). The differences of means were reported for measures that did not deviate from normality, and the differences of medians were reported for those that did. For these analyzes, the effects of interest were identified at an uncorrected threshold of *p* < 0.05.

### 2.4. Main effects of COVID-19 on connectivity

To assess the effects of COVID-19 on brain connectivity, a general linear model (GLM) estimated the effect of group for each network node, with covariates adjusting for age and sex. Standardized coefficient values were obtained, with bootstrapping (2000 iterations) to produce BSRs and 2-tailed *p*-value. For the main results, significantly altered connections were identified after adjusting for multiple comparisons over all network nodes, by applying a false discovery rate (FDR) threshold of 0.05. To further localize effects and evaluate the study hypotheses, the percentage of significant connections was calculated for each pair of the seven lobes: frontal, temporal, parietal, insular, limbic, occipital, subcortical. Lobe pairs were then identified where the percentage of significant connections exceeded those expected by chance alone, by randomly permuting the location of significant connections (5,000 iterations) and generating a *p*-value based on the fraction of permutations with a percentage value more extreme than the data. Significance was determined at an FDR threshold of 0.05, and pairs with significantly elevated percentage values were identified. In addition, for the set of significant connections, mean connectivity values were plotted for each participant in the control and COVID-19 groups. An overall coefficient of effect *b*, bootstrapped 95%CI, BSR and *p*-value were also reported based on these mean connectivity values.

### 2.5. Effects of clinical covariates on connectivity

Within the COVID-19 group, secondary analyzes examined whether functional connectivity was related to the total number of “ongoing” symptoms at the time of imaging, as an index of the severity of post-COVID outcome. Bootstrapped partial correlations were conducted, adjusting for age and sex, producing BSRs and 2-tailed *p*-values. Significant connections were again identified after adjusting for multiple comparisons over all network nodes, by applying an FDR threshold of 0.05. The percentage of significant connections present between each pair of lobes was again recorded, and significantly elevated percentages identified based on permutation testing as previously outlined, at an FDR threshold of 0.05. For the set of significant connections, mean connectivity values were plotted for each participant with COVID-19 against their symptom score, along with the control values for reference. An overall partial correlation coefficient, bootstrapped 95%CI and *p*-value were also reported per group, based on these mean connectivity values. As an alternative measure of clinical outcome, partial correlations were also obtained for the “combined” symptom count (ongoing and resolved); this measure is potentially more reflective of the cumulative effects of SARS-CoV-2 infection on brain function, rather than focusing solely on the effects of ongoing symptoms.

## 3. Results

**Figure 1:**
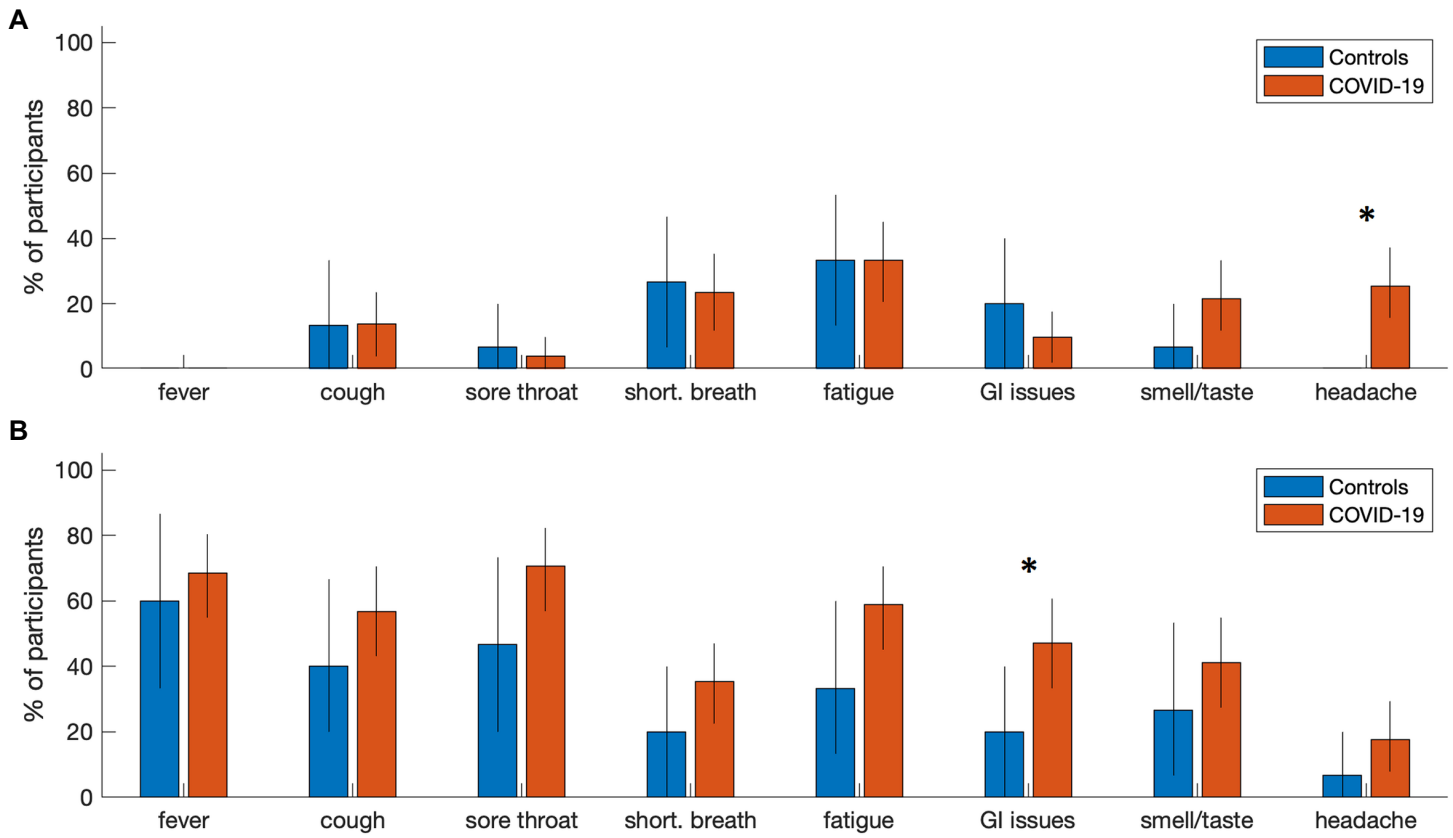
Symptom reporting rates for individuals with COVID-19 and controls. Bar plots summarize the percentage of participants reporting **(A)** ongoing symptoms and **(B)** resolved symptoms at time of imaging in each group. Error-bars denote bootstrapped 2-tailed 95% confidence intervals of the mean, and ‘*’ denotes group differences at *p* ≤ 0.05 (2-tailed).

At the time of analysis, 51 participants with COVID-19 and 15 controls had been recruited and scanned, with fMRI and T1-weighted anatomical imaging available. Participant demographics are summarized in Table 1 for COVID-19 and control groups. Both groups were comparable in age, proportion of male and female participants and years of education. There was substantial variability in imaging time post-onset for both cohorts, with both groups assessed a median of 4–5 months after their confirmed PCR test. In terms of symptoms, participants with COVID-19 tended to report a higher number of resolved symptoms and combined symptoms (resolved and ongoing) in comparison to controls, but not a higher number of ongoing symptoms on their own. Individual symptom reporting percentages are depicted in Figure 1. In comparison to the control group, the COVID-19 group showed an increased tendency to report ongoing headache (mean increase: 25.0%, 95%CI: [13.8%, 39.2%], BSR = 4.01, *p* < 0.001), and resolved gastrointestinal issues (27.1% [1.2%, 49.6%], BSR = 2.09, *p* = 0.036). All other symptom categories showed more limited group differences (all |BSR| ≤ 1.83, *p* ≥ 0.085), although the average reporting rates for all resolved symptoms are consistently higher in the COVID-19 group. For a detailed list of the specific symptoms reported in the “other” category, see Appendix 2. There was substantial reporting heterogeneity between individuals, although pain and body aches were most consistently identified in both the control (3 ongoing, 2 recovered) and COVID-19 (8 ongoing, 10 recovered) groups, and were most frequently localized to the chest. Issues related to cognition and memory were among the most consistently identified in the COVID-19 group alone (7 ongoing, 3 recovered).

**Figure 2:**
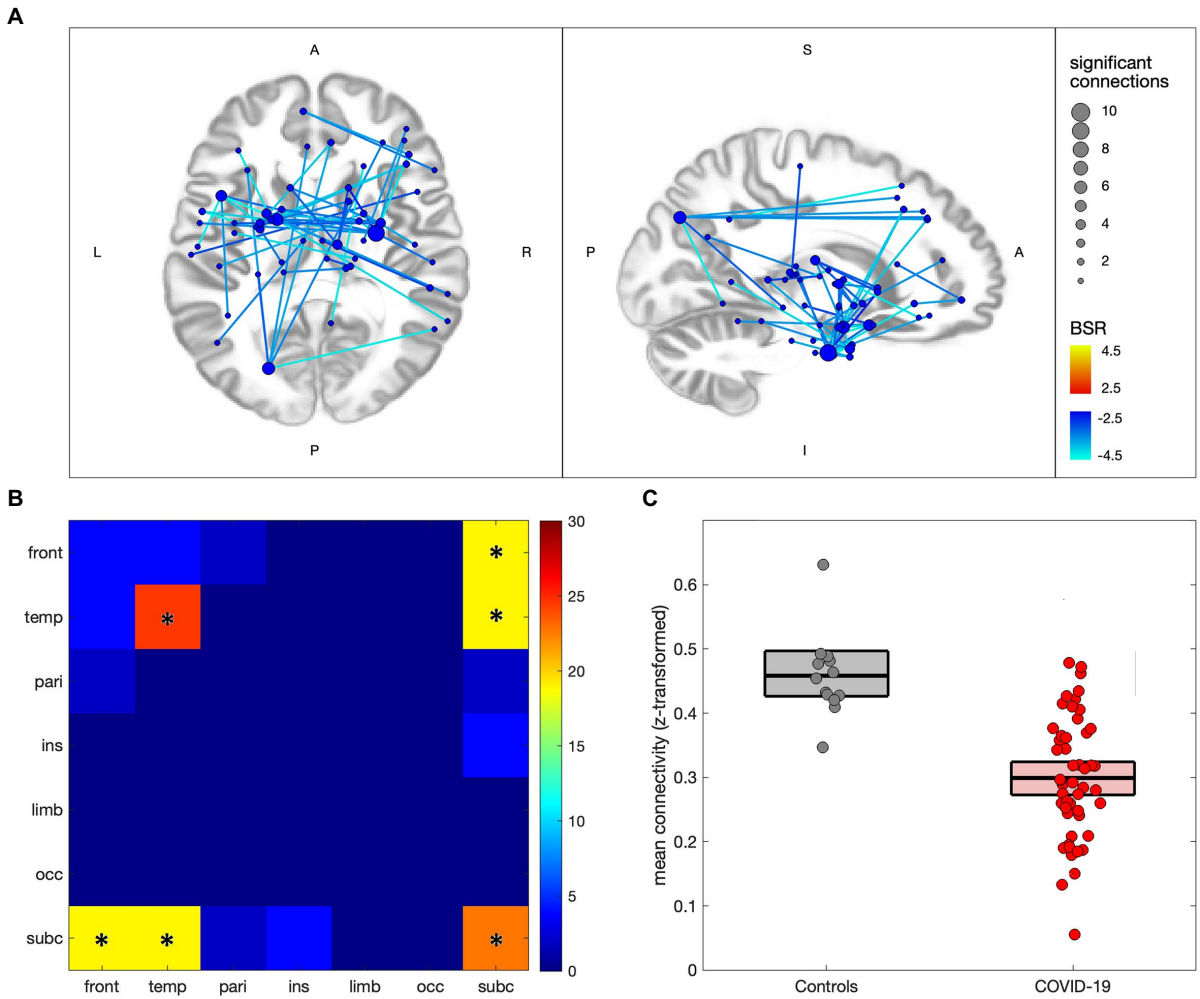
Altered network functional connectivity for individuals with COVID-19 relative to controls. **(A)** significant connections are depicted *via* lines connecting regions of interest (ROIs), with warm colors indicating increased connectivity for the COVID-19 group, and cool colors denoting decreased connectivity; line colors denote the strength of effect in terms of bootstrap ratio (BSR) and node sizes denote the number of significant connections. **(B)** heatmap showing the percentage of significant connections that occur between a given pair of lobes; a ‘*’ denotes significantly elevated connections for the lobe pair. **(C)** scatterplot showing mean connectivity values within regions of significant decrease, plotted for individuals in the COVID-19 and control groups; boxplots denote group means and 95%CIs of the mean.

Figure 2 compares the functional connectivity of participants with COVID-19 to controls, with 53/29,646 (0.18%) connections showing significant effects at an FDR of 0.05 (see Appendix 3, Supplementary Tables S2,S3 for details). In Figure 2A significant connections are shown to consist of uniform decreases in functional connectivity, with nodes mainly in subcortical and medial temporal regions, although frontal and parietal effects can also be seen. The greatest number of decreased connections are seen in the thalamus (22 connections total), parahippocampal gyri (19 connections), amygdala (14 connections), basal ganglia (10 connections) and superior temporal gyri (10 connections). Figure 2B shows the distribution of connections by lobe, with effects being mainly intra-temporal (*p* = 0.021), intra-subcortical (*p* < 0.001), temporal-subcortical (*p* = 0.001) and frontal-subcortical (*p* = 0.006). Figure 2C shows the difference in mean connectivity values for the participant groups, averaged over significant connections, with a mean difference of −0.159 ([−0.204, −0.119], BSR = -7.22, *p* < 0.001). Post-hoc analysis of the COVID-19 group found no significant associations of mean functional connectivity in these regions with years of education, days from symptom onset, days from testing, total number of ongoing symptoms, or the number of combined symptoms (ongoing and recovered), with all |BSR| ≤ 1.10 and *p* ≥ 0.277.

**Figure 3:**
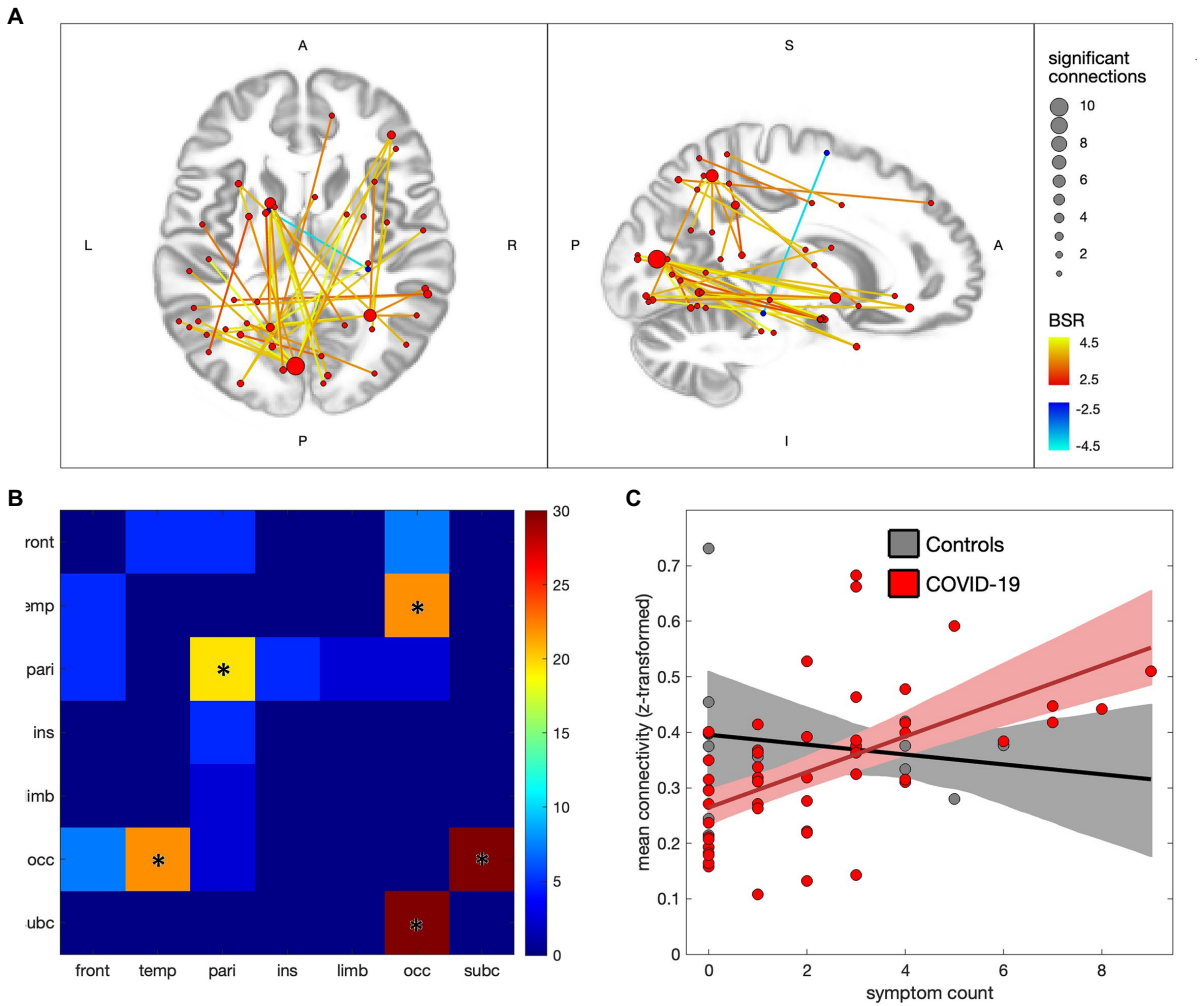
Effects of ongoing symptom count on functional connectivity for individuals with COVID-19. **(A)** significant connections are depicted by lines connecting regions of interest (ROIs), with warm colors indicating increased connectivity with greater symptom count, and cool colors denoting decreased connectivity; line colors denote the strength of effect in terms of bootstrap ratio (BSR) and node sizes denote the number of significant connections. **(B)** heatmap showing the percentage of significant connections that are between each pair of lobes; a ‘*’ denotes significantly elevated connections for the lobe pair. **(C)** scatterplot showing mean connectivity values within regions of significant increase, plotted against symptom count, for individuals in the COVID-19 and control groups; solid lines denote the lines of best fit and shaded areas denote the 95%CIs of the mean.

Figure 3 depicts the associations between ongoing symptom count and functional connectivity within the COVID-19 group, with 41/29,646 (0.14%) connections showing significant effects at an FDR of 0.05 (see Appendix 3, Supplementary Tables S4,S5 for details). In Figure 3A the significant connections are shown to consist of mainly positive associations with ongoing symptoms, with nodes in occipital, subcortical, temporal and parietal regions. The greatest number of positively associated connections are seen in the medioventral occipital cortex (21 connections total), inferior parietal lobule (11 connections), superior parietal lobule (8 connections) and basal ganglia (7 connections), while a single negatively associated connection is seen between the right parahippocampal gyrus and left superior frontal gyrus. Figure 3B shows the distribution of connections by lobe, with effects being mainly occipital-subcortical (*p* < 0.001), occipital-temporal (*p* < 0.001) and intra-parietal (*p* = 0.004). Figure 3C plots the mean connectivity values of significantly positive regions against ongoing symptom count for the COVID-19 group, along with the control group for comparison purposes. The partial correlation coefficient for the COVID-19 group was of moderate strength (ρ = 0.53 [0.38, 0.68], *p* < 0.001) whereas the controls showed near-zero correlations with symptoms (ρ = −0.06 [−0.76, 0.81], *p* = 0.960). Similar analyzes were also conducted on the combined symptom count (ongoing and recovered) and depicted in Appendix 4, with a comparable pattern of significantly affected brain regions and uniformly positive associations with symptom count.

## 4. Discussion

The present study investigated the neural correlates of COVID-19 in self-isolating individuals that were experiencing symptoms an average of 4–5 months post-infection. This is consistent with current PACS definitions (12, 13) and it is well outside of the 4-week window in which the SARS-CoV-2 virus is actively replicating (27). The study identified significant, uniformly decreased patterns of functional connectivity for the COVID-19 group in comparison to the control group. These decreases were mainly within and between temporal and subcortical regions, including the thalamus, parahippocampal gyri, amygdala, basal ganglia and superior temporal gyri. This suggests reduced functional integration of these areas, in terms of both local and long-range connections. Previous fMRI studies conducted outside of the infectious stage of COVID-19 have yielded mixed findings; studies of persistent olfactory dysfunction have reported increased connectivity within the olfactory network (28) and default-mode network (29), and greater connectivity between these networks (29). Another study of recovered individuals showed decreased connectivity within the temporal lobe and angular gyrus, but increased connectivity within the hippocampus (30). The present study findings suggest a distinct functional response associated with PACS, although given the methodological differences in the cited studies, any comparisons should be made with caution. In particular, the cited studies compared patients to healthy uninfected controls, making it unclear to what extent the effects are specific to COVID-19 and to what extent they represent a general response to viral infection.

The present study findings are partially congruent with a recent study of cerebral blood flow (CBF) conducted in this cohort, which also found long-term reductions in CBF in the prefrontal and subcortical nuclei of non-hospitalized COVID-19 patients (16). It is also aligned with a study comparing mild and severe forms of COVID-19, where the latter group had lower CBF in temporal regions (31). These findings suggest that vascular injury may contribute at least partially to the observed BOLD connectivity effects, potentially *via* endothelial infiltration of SARS-CoV-2 *via* angiotensin-converting enzyme 2 (ACE-2) receptors (32), or alternatively *via* inflammatory cytokine-mediated disruption of the blood–brain barrier. Other contributors may include altered neurometabolism, as PET studies in the acute and recovery phases of COVID-19 have found signs of hypo-metabolism relative to healthy controls (19, 20), including effects within the parahippocampal gyri. Similar hypometabolic effects were also seen in the amygdala, thalamus, and hippocampus among patients with PACS (33, 34). An additional contributor may be subtle decreases in cortical tissue (17, 18), e.g., due to astrocytic infections causing neuronal death or dysfunction (35).

The affected brain regions are noteworthy, given the commonly reported cognitive and behavioral sequelae of patients with COVID-19. The thalamus and basal ganglia have key integrative roles in sensory, motor and cognitive processes (36, 37), with the thalamus in particular mediating post-infection sickness behavior (38). In addition, the thalamus, amygdala and basal ganglia are implicated in pain processing (39), and may play a role in the headache symptoms seen in this study and others (40), along with pain and body ache reported in the “other” symptoms category. The amygdala has a key role in emotion regulation and stress response, which is relevant to post-COVID mood and mental health (41, 42). Neuroplastic alterations of the amygdala may further affect memory (43), while the parahippocampal gyri are critical hubs to memory formation (44); this is an area of concern, as memory impairments often occur after COVID-19 infection (45) and were reported in a subset of participants in this study, *via* the “other” symptoms category. Superior temporal involvement is also noteworthy, given its role in speech and language (46); although post-COVID language issues are not commonly reported, concerns have been raised due to prior studies of viral infection (47). The above cognitive and behavioral domains were not directly assessed in this study, which is a limitation that should be addressed in future research. However, the results provide preliminary evidence for brain areas and related functional domains that should be investigated in future research, with a particular focus on sensory processing, pain, emotion regulation, memory and language.

The present study also identified significant, mostly positive associations between functional connectivity and ongoing COVID-related symptoms. These effects mainly involved connections of subcortical and temporal regions to the occipital cortex, including the medioventral occipital cortex and basal ganglia. Intra-parietal effects were also observed, including the inferior and superior parietal lobules. The results suggest that elevated connectivity is associated with more severe post-COVID symptoms. Although understudied in this cohort, the relationship between symptoms and functional connectivity has been investigated in concussion, i.e., another form of diffuse neural injury. In this cohort, symptom severity is correlated with increased connectivity between functional networks in the early symptomatic phase of injury (48, 49) and reduced segregation between lobes of the brain at 6 months post-injury, for individuals with persistent post-concussion symptoms (50). These results are consistent with the positive associations between inter-lobe connectivity and symptom count seen in the COVID-19 group. The parietal effects seen in the COVID-19 group are also partly corroborated by a recent study of CBF within this cohort, which found elevated parietal blood flow in patients endorsing fatigue-related symptoms (16). The effects seen in the present study may stem from an aggregate of neural and systemic injury, the interoceptive mechanisms involved in detecting the consequences of injury, and/or adaptive neural mechanisms engaged in response to injury. The latter interpretation is consistent with a meta-analysis that reported hyper-connectivity of parietal, temporal and subcortical regions as part of a generalized response to neural injury (51). Nevertheless, further studies are needed to definitively establish the underlying mechanisms, and whether they constitute a pathological response or an adaptation to COVID-related neural injury.

This study significantly advances our understanding of PACS and its neural correlates, however, there are also some limitations to acknowledge. The present study focused on connectivity differences in symptomatic individuals with and without COVID-19 diagnosis. However, it is unclear (a) to what extent connectivity patterns in the control group are altered by infection and (b) to what extent connectivity patterns in the COVID-19 group are specific to PACS. To this end, future research should include additional control groups, consisting of non-infected individuals and COVID-infected individuals without persistent symptoms, respectively. The studied groups were also unbalanced, with COVID-19 having a substantially larger sample than controls. This leads to diminishing power gains, although the bootstrapping approach was chosen to partially mitigate these issues. Additionally, the larger COVID-19 sample enables the more precise quantification of within-group relationships between connectivity and symptoms. There was also variability in the time interval from symptom onset to MRI scan, although including this factor as a model covariate did not yield substantial effects, nor modify the main effect of interest, suggesting it has minimal impact on our conclusions. We also did not have information about variants of SARS-CoV-2 participants were infected with, although data collection ran from May 2020 to December 2021, with PCR testing an average of 4–5 prior, hence it is likely they were infected with earlier variants. Further work is needed to determine whether the neural effects of PACS depend significantly on the viral variant. Lastly, the analyzes of symptoms rely on self-report, which may be subject to a variety of reporting biases. The strength of the associations and congruency with related research supports the results, but further studies are needed to validate these findings, with emphasis on psychological factors that influence reporting habits, such as conscientiousness and neuroticism, with support from more objective neurocognitive testing.

Overall, the findings of this study suggest persistent alterations of the functional connectome after COVID-19 infection, for non-hospitalized individuals meeting criteria for PACS. These effects also appear to have circumscribed localization, mainly to temporal and subcortical regions. Furthermore, there is a distinct pattern of occipital, temporal, subcortical and parietal connectivity associated with severity of PACS symptoms. These results indicate that physiological recovery from COVID-19 may extend well beyond the resolution of acute symptoms, with the persistence of post-COVID brain changes raising particular concerns about the cumulative effects of repeated infection on brain function (52). The results further suggest a distinct neural response related to severity of PACS symptoms, helping to better clarify the neural mechanisms that underlie heterogeneous patient outcomes after infection.

## Data availability statement

The original contributions presented in the study are publicly available. This data can be found here: Figshare, https://doi.org/10.6084/m9.figshare.21797735.

## Ethics statement

The studies involving human participants were reviewed and approved by Sunnybrook Health Sciences Centre ethics board. The patients/participants provided their written informed consent to participate in this study.

## Author contributions

ER, JC, AG, AS, XJ, FG, ZL, AJ, MM, MG, JR, BL, IC, RF, CH, SB, BM, and SG: study design. XJ, ER, ZL, AJ, SG, and BM: data collection. NC, ER, JC, AG, AS, XJ, FG, ZL, AJ, MM, MG, JR, BL, IC, RF, CH, SB, BM, SG, and TS: data analysis and interpretation. NC, SG, and TS: manuscript writing. All authors contributed to the article and approved the submitted version.

## Funding

This study is funded in part by the Sunnybrook Foundation, the SB Centre for Brain Resilience & Recovery, a Canadian Institutes of Health Research (CIHR) Project Grant (165981), and a CIHR Operating Grant on Emerging COVID-19 Research Gaps and Priorities (177756).

## Conflict of interest

SB reports payments for contract research to her institution from GE Healthcare, Eli Lilly and Company, Biogen, Genentech, Optina Diagnostics, and Roche; consulting fees and payments related to an advisory board from Roche; and payments related to an advisory board, a speaker panel, talks, and an educational session from Biogen. There were peer-reviewed grants to her institution from the Ontario Brain Institute, Canadian Institutes of Health Research, Leducq Foundation, Heart and Stroke Foundation of Canada, National Institutes of Health, Alzheimer’s Drug Discovery Foundation, Brain Canada, Weston Brain Institute, Canadian Partnership for Stroke Recovery, Canadian Foundation for Innovation, Focused Ultrasound Foundation, Alzheimer’s Association US, Department of National Defense, Montreal Medical International Kuwait, Queen’s University, Compute Canada Resources for Research Groups, CANARIE, and Networks of Centres of Excellence of Canada. She has participated on a data safety monitoring board or advisory board for the Conference Board of Canada, World Dementia Council, and University of Rochester. She has contributed to the mission and scientific leadership of the Small Vessel VCID Biomarker Validation Consortium, National Institute of Neurological Disorders and Stroke.

The remaining authors declare that the research was conducted in the absence of any commercial or financial relationships that could be construed as a potential conflict of interest.

## Publisher’s note

All claims expressed in this article are solely those of the authors and do not necessarily represent those of their affiliated organizations, or those of the publisher, the editors and the reviewers. Any product that may be evaluated in this article, or claim that may be made by its manufacturer, is not guaranteed or endorsed by the publisher.
