# Supplementary material for: Effects of post-acute COVID-19 syndrome on the functional brain networks of non-hospitalized individuals
Source: Front Neurol. 2023 Mar 27;14:1136408. doi: 10.3389/fneur.2023.1136408 (PMC10083436; doi:10.3389/fneur.2023.1136408)
Supplement: Supplementary file 2 [file Data_Sheet_2.docx]

**Appendix-2: self-reported symptoms in the “other” category**

**Methods**

The symptom questionnaire administered to study participants included an “other” category which allowed them to report any post-infection symptoms that did not fit within the initial eight categories (i.e., fever, cough, sore throat, shortness of breath, fatigue, gastrointestinal issues, problems with smell/taste, headache). For participants reporting in the “other” category, the symptoms were collected, checked for consistent labelling and presented in Table S1 below, with participants ordered by decreasing number of symptoms in this category. A qualitative summary of the most prevalent issues is provided.

**Results**

Table S1 below shows substantial inter-individual heterogeneity of reporting within the “other” category, for both the control and COVID-19 groups. Pain was the most consistently reported symptom type, reported in both the control group (2 ongoing, 1 recovered) and COVID-19 group (6 ongoing, 5 recovered), with localization to the chest being most common. Body aches were also reported in both the control group (1 ongoing) and COVID-19 group (2 ongoing, 5 recovered). Also common are reports of dizziness, vertigo and light-headedness for both the control group (1 ongoing) and COVID-19 group (4 ongoing). Symptoms specific to the COVID-19 group include complaints of cognitive issues, brain fog, confusion (4 ongoing, 3 recovered) and, relatedly, issues with memory (3 ongoing). Other symptoms are less consistently reported and cover a range of categories, including congestion (chest congestion, sinus congestion, phlegm, chest clearing), vision issues (including eye fatigue), signs of dysautonomia (high blood pressure, elevated heart rate, heart palpitations) and issues related to headache (light sensitivity, nausea, migraine).

**Table S1**: list of symptoms reported in the “other” category for each group, by participant. This included 6/15 participants from the control group and 38/51 from the COVID-19 group. Ongoing symptoms are shaded in dark grey, recovered symptoms are shaded in light grey.

|  |  | **symptom-1** | **symptom-2** | **symptom-3** | **symptom-4** | **symptom-5** | **symptom-6** |
| --- | --- | --- | --- | --- | --- | --- | --- |
| **control** | 1 | swollen lymph nodes | light headed | heart palpitations | burning lungs |  |  |
|  | 2 | elevated heart rate | head pressure | arm/back pain | chest pain |  |  |
|  | 3 | body pain | facial numbness/pain | tingling |  |  |  |
|  | 4 | chest pain/pressure | muscle ache | arm pain/weakness |  |  |  |
|  | 5 | elevated heart rate | chest congestion |  |  |  |  |
|  | 6 | sinus congestion | post-nasal drip |  |  |  |  |
| **COVID-19** | 1 | memory issues | high blood pressure | elevated heart rate | chest pain | hair loss | rash |
|  | 2 | hearing issues | tingling | hand tremor | eye fatigue | foot numbness |  |
|  | 3 | neck/chest pain | upper body pain (bone) | upper body pain (muscle) | elevated heart rate (night time) | foot itch |  |
|  | 4 | brain fog | dizziness | joint pain | memory issues |  |  |
|  | 5 | heart palpitations | muscle spasm (ear) | sinus pressure/pain | light sensitivity |  |  |
|  | 6 | chills | phlegm | body ache | chest clearing |  |  |
|  | 7 | chills | cold sweat | back ache |  |  |  |
|  | 8 | dizziness | vertigo | exertional malaise |  |  |  |
|  | 9 | chest tightness | ear ache | spleen pain |  |  |  |
|  | 10 | vertigo | confusion | face tingling |  |  |  |
|  | 11 | memory issues | swollen brain feeling | swelling in extremities |  |  |  |
|  | 12 | brain fog | light sensitivity | bronchitis |  |  |  |
|  | 13 | brain fog | side pain |  |  |  |  |
|  | 14 | sinus pain | ear pain |  |  |  |  |
|  | 15 | dizziness | chest pressure |  |  |  |  |
|  | 16 | migraine | sinus congestion |  |  |  |  |
|  | 17 | chest pain | back pain |  |  |  |  |
|  | 18 | chills | light sensitivity |  |  |  |  |
|  | 19 | vomitting | vision issues |  |  |  |  |
|  | 20 | high blood pressure |  |  |  |  |  |
|  | 21 | high blood pressure |  |  |  |  |  |
|  | 22 | post-nasal drip |  |  |  |  |  |
|  | 23 | chest pain |  |  |  |  |  |
|  | 24 | body ache |  |  |  |  |  |
|  | 25 | slow thinking |  |  |  |  |  |
|  | 26 | migraine |  |  |  |  |  |
|  | 27 | thyroid swelling |  |  |  |  |  |
|  | 28 | cold sweat |  |  |  |  |  |
|  | 29 | cognitive issues |  |  |  |  |  |
|  | 30 | cognitive issues |  |  |  |  |  |
|  | 31 | chest pain |  |  |  |  |  |
|  | 32 | Body aches |  |  |  |  |  |
|  | 33 | cognitive issues |  |  |  |  |  |
|  | 34 | body ache |  |  |  |  |  |
|  | 35 | Chest pain |  |  |  |  |  |
|  | 36 | Sore muscles |  |  |  |  |  |
|  | 37 | nausea |  |  |  |  |  |
|  | 38 | muscle spasm |  |  |  |  |  |
