# Supplementary material for: Effects of post-acute COVID-19 syndrome on the functional brain networks of non-hospitalized individuals
Source: Front Neurol. 2023 Mar 27;14:1136408. doi: 10.3389/fneur.2023.1136408 (PMC10083436; doi:10.3389/fneur.2023.1136408)
Supplement: Supplementary file 3 [file Data_Sheet_3.DOCX]

**Appendix-3: supplemental tables**

**Table S2**: pairwise connections showing significant differences for COVID-19 patients relative to controls. Brain regions are from the Brainnetome atlas, with centers of mass (CoMs) given in Montréal Neurological Institute (MNI) space coordinates. Bootstrap ratio values (BSRs) provide z-scored standardized measures of effect size.

|  | region 1 | CoM 1 | | | region 2 | CoM 2 | | | BSR |
| --- | --- | --- | --- | --- | --- | --- | --- | --- | --- |
| 1 | Amygdala_L_(lAmyg) | -16 | -2 | -20 | Superior_temporal_gyrus_L_(A38l) | -44 | 10 | -20 | -4.64 |
| 2 | Parahippocampal_gyrus_L_(A35/36c) | -26 | -8 | -34 | Middle_temporal_gyrus_L_(A21r) | -52 | 2 | -28 | -4.44 |
| 3 | Inferior_temporal_gyrus_L_(A20il) | -56 | -16 | -28 | Superior_temporal_gyrus_L_(A38l) | -44 | 10 | -20 | -4.37 |
| 4 | Amygdala_L_(mAmyg) | -22 | -76 | 34 | Inferior_temporal_gyrus_R_(A37vl) | 56 | -58 | -8 | -4.35 |
| 5 | Postcentral_gyrus_R_(A1/2/3ulhf) | 8 | -56 | 34 | Superior_frontal_gyrus_R_(A8dl) | 22 | 26 | 52 | -4.34 |
| 6 | Basal_ganglia_L_(NAC) | -22 | -2 | 4 | Orbital_gyrus_L_(A12/47o) | -34 | 34 | -16 | -4.30 |
| 7 | Parahippocampal_gyrus_R_(A35/36c) | 26 | -8 | -34 | Orbital_gyrus_R_(A12/47l) | 44 | 32 | -8 | -4.29 |
| 8 | Thalamus_R_(rTtha) | 20 | -22 | 4 | Insular_gyrus_L_(dId) | -38 | -8 | 8 | -4.24 |
| 9 | Parahippocampal_gyrus_L_(TH) | -22 | 2 | -32 | Middle_temporal_gyrus_R_(A37dl) | 62 | -52 | 2 | -4.21 |
| 10 | Amygdala_R_(lAmyg) | 20 | -2 | -20 | Parahippocampal_gyrus_R_(A35/36c) | 26 | -8 | -34 | -4.19 |
| 11 | Hippocampus_L_(rHipp) | -26 | -4 | -20 | Superior_temporal_gyrus_L_(A38l) | -44 | 10 | -20 | -4.16 |
| 12 | Parahippocampal_gyrus_R_(A35/36c) | 26 | -8 | -34 | Middle_temporal_gyrus_L_(A21r) | -52 | 2 | -28 | -4.15 |
| 13 | Hippocampus_L_(rHipp) | -26 | -4 | -20 | Middle_frontal_gyrus_R_(A8vl) | 40 | 26 | 40 | -4.09 |
| 14 | Hippocampus_L_(rHipp) | -26 | -4 | -20 | Superior_frontal_gyrus_R_(A9m) | 4 | 38 | 34 | -4.06 |
| 15 | Amygdala_R_(lAmyg) | 20 | -2 | -20 | Parahippocampal_gyrus_L_(TH) | -22 | 2 | -32 | -4.04 |
| 16 | Amygdala_L_(lAmyg) | -16 | -2 | -20 | Middle_frontal_gyrus_R_(A8vl) | 40 | 26 | 40 | -3.98 |
| 17 | Parahippocampal_gyrus_R_(TH) | 22 | 2 | -34 | Inferior_temporal_gyrus_L_(A20iv) | -44 | -22 | -28 | -3.84 |
| 18 | Amygdala_L_(mAmyg) | -22 | -76 | 34 | Superior_frontal_gyrus_R_(A9m) | 4 | 38 | 34 | -3.82 |
| 19 | Parahippocampal_gyrus_R_(TH) | 22 | 2 | -34 | Inferior_temporal_gyrus_R_(A20iv) | 46 | -14 | -32 | -3.81 |
| 20 | Post_super_temp_sulcus_R_(cpSTS) | 52 | -38 | 4 | Parahippocampal_gyrus_L_(TH) | -22 | 2 | -32 | -3.78 |
| 21 | Orbital_gyrus_R_(A12/47l) | 44 | 32 | -8 | Orbital_gyrus_L_(A14m) | -8 | 52 | -8 | -3.76 |
| 22 | Parahippocampal_gyrus_R_(A35/36c) | 26 | -8 | -34 | Orbital_gyrus_R_(A12/47o) | 40 | 38 | -14 | -3.75 |
| 23 | Amygdala_L_(mAmyg) | -22 | -76 | 34 | Middle_frontal_gyrus_L_(A8vl) | -34 | 22 | 46 | -3.70 |
| 24 | Amygdala_L_(mAmyg) | -22 | -76 | 34 | Superior_frontal_gyrus_L_(A9m) | -4 | 34 | 38 | -3.69 |
| 25 | Thalamus_R_(IPFtha) | 10 | -16 | 14 | Amygdala_L_(lAmyg) | -16 | -2 | -20 | -3.68 |
| 26 | Parahippocampal_gyrus_L_(A35/36r) | -40 | -50 | -16 | Superior_temporal_gyrus_L_(A38l) | -44 | 10 | -20 | -3.68 |
| 27 | Hippocampus_R_(cHipp) | 22 | -14 | -20 | Middle_frontal_gyrus_R_(A9/46v) | 40 | 44 | 14 | -3.65 |
| 28 | Thalamus_R_(mPFtha) | 28 | -2 | 2 | Basal_ganglia_L_(GP) | -14 | 14 | -2 | -3.60 |
| 29 | Parahippocampal_gyrus_L_(TH) | -22 | 2 | -32 | Inferior_temporal_gyrus_R_(A20cl) | 62 | -40 | -16 | -3.59 |
| 30 | Thalamus_R_(cTtha) | 14 | -28 | 8 | Thalamus_L_(cTtha) | -16 | -28 | 4 | -3.57 |
| 31 | Parahippocampal_gyrus_R_(A35/36c) | 26 | -8 | -34 | Superior_temporal_gyrus_L_(A22r) | -56 | -4 | -10 | -3.56 |
| 32 | Amygdala_L_(mAmyg) | -22 | -76 | 34 | Middle_frontal_gyrus_R_(A9/46d) | 32 | 38 | 34 | -3.54 |
| 33 | Amygdala_L_(lAmyg) | -16 | -2 | -20 | Parahippocampal_gyrus_R_(A35/36c) | 26 | -8 | -34 | -3.54 |
| 34 | Thalamus_L_(mPMtha) | -8 | -14 | 4 | Inferior_parietal_lobule_L_(A40rv) | -46 | -64 | 26 | -3.52 |
| 35 | Basal_ganglia_R_(vmPu) | 14 | 8 | -8 | Amygdala_L_(lAmyg) | -16 | -2 | -20 | -3.49 |
| 36 | Thalamus_L_(Otha) | -16 | -22 | 8 | Parahippocampal_gyrus_L_(A35/36c) | -26 | -8 | -34 | -3.45 |
| 37 | Thalamus_R_(mPFtha) | 28 | -2 | 2 | Amygdala_L_(lAmyg) | -16 | -2 | -20 | -3.43 |
| 38 | Thalamus_R_(mPFtha) | 28 | -2 | 2 | Basal_ganglia_L_(vmPu) | -16 | 4 | -8 | -3.43 |
| 39 | Orbital_gyrus_L_(A14m) | -8 | 52 | -8 | Inferior_frontal_gyrus_R_(A45c) | 56 | 22 | 10 | -3.43 |
| 40 | Parahippocampal_gyrus_R_(A35/36c) | 26 | -8 | -34 | Superior_temporal_gyrus_R_(A41/42) | 52 | -26 | 10 | -3.41 |
| 41 | Parahippocampal_gyrus_R_(A35/36c) | 26 | -8 | -34 | Superior_temporal_gyrus_L_(A38l) | -44 | 10 | -20 | -3.34 |
| 42 | Basal_ganglia_L_(NAC) | -22 | -2 | 4 | Parahippocampal_gyrus_L_(A35/36c) | -26 | -8 | -34 | -3.30 |
| 43 | Thalamus_L_(mPFtha) | -28 | -4 | 2 | Basal_ganglia_L_(vmPu) | -16 | 4 | -8 | -3.30 |
| 44 | Thalamus_R_(IPFtha) | 10 | -16 | 14 | Basal_ganglia_L_(GP) | -14 | 14 | -2 | -3.26 |
| 45 | Thalamus_R_(cTtha) | 14 | -28 | 8 | Thalamus_R_(Otha) | 14 | -22 | 8 | -3.26 |
| 46 | Basal_ganglia_L_(vCa) | -28 | -28 | -10 | Amygdala_L_(mAmyg) | -22 | -76 | 34 | -3.25 |
| 47 | Thalamus_R_(IPFtha) | 10 | -16 | 14 | Basal_ganglia_R_(vmPu) | 14 | 8 | -8 | -3.24 |
| 48 | Thalamus_R_(mPFtha) | 28 | -2 | 2 | Insular_gyrus_L_(dIg) | -40 | -4 | -10 | -3.23 |
| 49 | Thalamus_R_(Otha) | 14 | -22 | 8 | Paracentral_lobule_R_(A4ll) | 4 | -22 | 62 | -3.13 |
| 50 | Thalamus_R_(IPFtha) | 10 | -16 | 14 | Basal_ganglia_R_(GP) | 16 | 14 | -2 | -3.12 |
| 51 | Middle_temporal_gyrus_L_(aSTS) | -58 | -20 | -10 | Superior_temporal_gyrus_R_(A38l) | 46 | 14 | -20 | -3.09 |
| 52 | Thalamus_R_(PPtha) | 4 | -14 | 4 | Post_super_temp_sulcus_R_(cpSTS) | 52 | -38 | 4 | -3.08 |
| 53 | Basal_ganglia_R_(GP) | 16 | 14 | -2 | Parahippocampal_gyrus_R_(A35/36c) | 26 | -8 | -34 | -2.76 |

**Table S3**: list of all regions showing significant differences for COVID-19 patients relative to controls. Brain regions are from the Brainnettome atlas, with centers of mass (CoMs) given in Montréal Neurological Institute (MNI) space coordinates. Number of significant connections is identified, along with the average bootstrap ratio values (BSRs) for these connections.

|  | region | CoM | | | Number of connections | mean BSR |
| --- | --- | --- | --- | --- | --- | --- |
| 1 | Parahippocampal_gyrus_R_(A35/36c) | 26 | -8 | -34 | 9 | -3.67 |
| 2 | Amygdala_L_(mAmyg) | -22 | -76 | 34 | 6 | -3.73 |
| 3 | Amygdala_L_(lAmyg) | -16 | -2 | -20 | 6 | -3.79 |
| 4 | Superior_temporal_gyrus_L_(A38l) | -44 | 10 | -20 | 5 | -4.04 |
| 5 | Parahippocampal_gyrus_L_(TH) | -22 | 2 | -32 | 4 | -3.90 |
| 6 | Thalamus_R_(mPFtha) | 28 | -2 | 2 | 4 | -3.42 |
| 7 | Thalamus_R_(IPFtha) | 10 | -16 | 14 | 4 | -3.33 |
| 8 | Parahippocampal_gyrus_L_(A35/36c) | -26 | -8 | -34 | 3 | -3.73 |
| 9 | Hippocampus_L_(rHipp) | -26 | -4 | -20 | 3 | -4.10 |
| 10 | Superior_frontal_gyrus_R_(A9m) | 4 | 38 | 34 | 2 | -3.94 |
| 11 | Middle_frontal_gyrus_R_(A8vl) | 40 | 26 | 40 | 2 | -4.04 |
| 12 | Orbital_gyrus_L_(A14m) | -8 | 52 | -8 | 2 | -3.60 |
| 13 | Orbital_gyrus_R_(A12/47l) | 44 | 32 | -8 | 2 | -4.02 |
| 14 | Middle_temporal_gyrus_L_(A21r) | -52 | 2 | -28 | 2 | -4.30 |
| 15 | Parahippocampal_gyrus_R_(TH) | 22 | 2 | -34 | 2 | -3.83 |
| 16 | Post_super_temp_sulcus_R_(cpSTS) | 52 | -38 | 4 | 2 | -3.43 |
| 17 | Amygdala_R_(lAmyg) | 20 | -2 | -20 | 2 | -4.12 |
| 18 | Basal_ganglia_L_(GP) | -14 | 14 | -2 | 2 | -3.43 |
| 19 | Basal_ganglia_R_(GP) | 16 | 14 | -2 | 2 | -2.94 |
| 20 | Basal_ganglia_L_(NAC) | -22 | -2 | 4 | 2 | -3.80 |
| 21 | Basal_ganglia_L_(vmPu) | -16 | 4 | -8 | 2 | -3.37 |
| 22 | Basal_ganglia_R_(vmPu) | 14 | 8 | -8 | 2 | -3.36 |
| 23 | Thalamus_R_(Otha) | 14 | -22 | 8 | 2 | -3.19 |
| 24 | Thalamus_R_(cTtha) | 14 | -28 | 8 | 2 | -3.41 |
| 25 | Superior_frontal_gyrus_R_(A8dl) | 22 | 26 | 52 | 1 | -4.34 |
| 26 | Superior_frontal_gyrus_L_(A9m) | -4 | 34 | 38 | 1 | -3.69 |
| 27 | Middle_frontal_gyrus_R_(A9/46d) | 32 | 38 | 34 | 1 | -3.54 |
| 28 | Middle_frontal_gyrus_R_(A9/46v) | 40 | 44 | 14 | 1 | -3.65 |
| 29 | Middle_frontal_gyrus_L_(A8vl) | -34 | 22 | 46 | 1 | -3.70 |
| 30 | Inferior_frontal_gyrus_R_(A45c) | 56 | 22 | 10 | 1 | -3.43 |
| 31 | Orbital_gyrus_L_(A12/47o) | -34 | 34 | -16 | 1 | -4.30 |
| 32 | Orbital_gyrus_R_(A12/47o) | 40 | 38 | -14 | 1 | -3.75 |
| 33 | Paracentral_lobule_R_(A4ll) | 4 | -22 | 62 | 1 | -3.13 |
| 34 | Superior_temporal_gyrus_R_(A41/42) | 52 | -26 | 10 | 1 | -3.41 |
| 35 | Superior_temporal_gyrus_R_(A38l) | 46 | 14 | -20 | 1 | -3.09 |
| 36 | Superior_temporal_gyrus_L_(A22r) | -56 | -4 | -10 | 1 | -3.56 |
| 37 | Middle_temporal_gyrus_R_(A37dl) | 62 | -52 | 2 | 1 | -4.21 |
| 38 | Middle_temporal_gyrus_L_(aSTS) | -58 | -20 | -10 | 1 | -3.09 |
| 39 | Inferior_temporal_gyrus_L_(A20iv) | -44 | -22 | -28 | 1 | -3.84 |
| 40 | Inferior_temporal_gyrus_R_(A20iv) | 46 | -14 | -32 | 1 | -3.81 |
| 41 | Inferior_temporal_gyrus_L_(A20il) | -56 | -16 | -28 | 1 | -4.37 |
| 42 | Inferior_temporal_gyrus_R_(A37vl) | 56 | -58 | -8 | 1 | -4.35 |
| 43 | Inferior_temporal_gyrus_R_(A20cl) | 62 | -40 | -16 | 1 | -3.59 |
| 44 | Parahippocampal_gyrus_L_(A35/36r) | -40 | -50 | -16 | 1 | -3.68 |
| 45 | Inferior_parietal_lobule_L_(A40rv) | -46 | -64 | 26 | 1 | -3.52 |
| 46 | Postcentral_gyrus_R_(A1/2/3ulhf) | 8 | -56 | 34 | 1 | -4.34 |
| 47 | Insular_gyrus_L_(dIg) | -40 | -4 | -10 | 1 | -3.23 |
| 48 | Insular_gyrus_L_(dId) | -38 | -8 | 8 | 1 | -4.24 |
| 49 | Hippocampus_R_(cHipp) | 22 | -14 | -20 | 1 | -3.65 |
| 50 | Basal_ganglia_L_(vCa) | -28 | -28 | -10 | 1 | -3.25 |
| 51 | Thalamus_L_(mPFtha) | -28 | -4 | 2 | 1 | -3.30 |
| 52 | Thalamus_L_(mPMtha) | -8 | -14 | 4 | 1 | -3.52 |
| 53 | Thalamus_R_(rTtha) | 20 | -22 | 4 | 1 | -4.24 |
| 54 | Thalamus_R_(PPtha) | 4 | -14 | 4 | 1 | -3.08 |
| 55 | Thalamus_L_(Otha) | -16 | -22 | 8 | 1 | -3.45 |
| 56 | Thalamus_L_(cTtha) | -16 | -28 | 4 | 1 | -3.57 |

**Table S4**: pairwise connections showing significant effects of ongoing symptom count for COVID-19 patients. Brain regions are from the Brainnetome atlas, with centers of mass (CoMs) given in Montréal Neurological Institute (MNI) space coordinates. Bootstrap ratio values (BSRs) provide z-scored standardized measures of effect size.

|  | region 1 | CoM 1 | | | region 2 | CoM 2 | | | BSR |
| --- | --- | --- | --- | --- | --- | --- | --- | --- | --- |
| 1 | Amygdala_R_(lAmyg) | 20 | -2 | -20 | Mediovent_occipital_cortex_L_(cCunG) | -4 | -80 | 10 | 4.90 |
| 2 | Mediovent_occipital_cortex_L_(cCunG) | -4 | -80 | 10 | Inferior_temporal_gyrus_L_(A20iv) | -44 | -22 | -28 | 4.78 |
| 3 | Mediovent_occipital_cortex_L_(cCunG) | -4 | -80 | 10 | Fusiform_gyrus_L_(A37lv) | -32 | -64 | -14 | 4.64 |
| 4 | Basal_ganglia_R_(vCa) | 28 | -28 | -10 | Mediovent_occipital_cortex_L_(vmPOS) | -16 | -62 | -8 | 4.39 |
| 5 | Inferior_parietal_lobule_L_(A40rd) | -38 | -62 | 46 | Inferior_parietal_lobule_R_(A39c) | 32 | -56 | 52 | 4.37 |
| 6 | Basal_ganglia_L_(vmPu) | -16 | 4 | -8 | Mediovent_occipital_cortex_R_(rLinG) | 8 | -88 | 14 | 4.36 |
| 7 | Amygdala_L_(lAmyg) | -16 | -2 | -20 | Mediovent_occipital_cortex_L_(rCunG) | -10 | -82 | -10 | 4.35 |
| 8 | Parahippocampal_gyrus_R_(A28/34) | 28 | -28 | -20 | Superior_frontal_gyrus_L_(A6dl) | -16 | -2 | 64 | -4.25 |
| 9 | Basal_ganglia_L_(vmPu) | -16 | 4 | -8 | Mediovent_occipital_cortex_R_(rCunG) | 10 | -86 | -8 | 4.23 |
| 10 | Mediovent_occipital_cortex_L_(cCunG) | -4 | -80 | 10 | Superior_temporal_gyrus_L_(A38m) | -32 | 14 | -34 | 4.15 |
| 11 | Mediovent_occipital_cortex_L_(cCunG) | -4 | -80 | 10 | Inferior_temporal_gyrus_L_(A37elv) | -50 | -58 | -14 | 4.10 |
| 12 | Basal_ganglia_L_(vmPu) | -16 | 4 | -8 | Mediovent_occipital_cortex_L_(vmPOS) | -16 | -62 | -8 | 4.09 |
| 13 | Insular_gyrus_R_(dId) | 40 | -8 | 8 | Inferior_parietal_lobule_R_(A39c) | 32 | -56 | 52 | 4.09 |
| 14 | Postcentral_gyrus_R_(A2) | 56 | -10 | 14 | Superior_parietal_lobule_L_(A5l) | -16 | -70 | 52 | 4.08 |
| 15 | Mediovent_occipital_cortex_L_(cCunG) | -4 | -80 | 10 | Middle_temporal_gyrus_L_(A37dl) | -58 | -58 | 4 | 4.07 |
| 16 | Inferior_parietal_lobule_L_(A40rv) | -46 | -64 | 26 | Inferior_parietal_lobule_R_(A39c) | 32 | -56 | 52 | 4.05 |
| 17 | Fusiform_gyrus_R_(A37lv) | 32 | -62 | -14 | Orbital_gyrus_R_(A12/47o) | 40 | 38 | -14 | 4.05 |
| 18 | Mediovent_occipital_cortex_L_(cCunG) | -4 | -80 | 10 | Orbital_gyrus_R_(A12/47o) | 40 | 38 | -14 | 4.04 |
| 19 | Lateral_occipital_cortex_L_(V5/MT+) | -32 | -88 | 10 | Orbital_gyrus_R_(A12/47l) | 44 | 32 | -8 | 4.03 |
| 20 | Mediovent_occipital_cortex_L_(cCunG) | -4 | -80 | 10 | Inferior_temporal_gyrus_L_(A37vl) | -56 | -62 | -8 | 4.03 |
| 21 | Lateral_occipital_cortex_L_(V5/MT+) | -32 | -88 | 10 | Orbital_gyrus_R_(A12/47o) | 40 | 38 | -14 | 3.99 |
| 22 | Basal_ganglia_L_(dlPu) | -14 | 2 | 16 | Mediovent_occipital_cortex_R_(rCunG) | 10 | -86 | -8 | 3.92 |
| 23 | Lateral_occipital_cortex_R_(OPC) | 50 | -70 | -2 | Superior_temporal_gyrus_L_(A38m) | -32 | 14 | -34 | 3.92 |
| 24 | Inferior_parietal_lobule_R_(A39rv) | 58 | -44 | 38 | Superior_parietal_lobule_L_(A7r) | -52 | -50 | 10 | 3.90 |
| 25 | Mediovent_occipital_cortex_L_(cCunG) | -4 | -80 | 10 | Fusiform_gyrus_L_(A20rv) | -56 | -32 | -28 | 3.88 |
| 26 | Cingulate_gyrus_R_(A23c) | 4 | 4 | 38 | Superior_parietal_lobule_L_(A7ip) | -22 | -46 | 64 | 3.81 |
| 27 | Inferior_parietal_lobule_R_(A40rv) | 52 | -56 | 26 | Inferior_parietal_lobule_R_(A39c) | 32 | -56 | 52 | 3.68 |
| 28 | Hippocampus_L_(rHipp) | -26 | -4 | -20 | Mediovent_occipital_cortex_L_(cCunG) | -4 | -80 | 10 | 3.66 |
| 29 | Lateral_occipital_cortex_R_(msOccG) | 32 | -86 | -10 | Inferior_parietal_lobule_L_(A39c) | -28 | -58 | 52 | 3.64 |
| 30 | Hippocampus_R_(rHipp) | 28 | -4 | -20 | Mediovent_occipital_cortex_L_(cCunG) | -4 | -80 | 10 | 3.62 |
| 31 | Inferior_parietal_lobule_R_(A39c) | 32 | -56 | 52 | Superior_parietal_lobule_R_(A7r) | 56 | -40 | 14 | 3.61 |
| 32 | Basal_ganglia_L_(vmPu) | -16 | 4 | -8 | Mediovent_occipital_cortex_L_(rCunG) | -10 | -82 | -10 | 3.58 |
| 33 | Basal_ganglia_L_(vmPu) | -16 | 4 | -8 | Mediovent_occipital_cortex_R_(vmPOS) | 20 | -62 | -8 | 3.53 |
| 34 | Superior_parietal_lobule_L_(A5l) | -16 | -70 | 52 | Superior_frontal_gyrus_R_(A9l) | 14 | 50 | 40 | 3.52 |
| 35 | Insular_gyrus_R_(dIa) | 32 | 14 | -14 | Inferior_parietal_lobule_R_(A39c) | 32 | -56 | 52 | 3.51 |
| 36 | Superior_parietal_lobule_L_(A7c) | -16 | -58 | 62 | Precentral_gyrus_L_(A4hf) | -50 | -8 | 40 | 3.44 |
| 37 | Inferior_parietal_lobule_R_(A39rv) | 58 | -44 | 38 | Superior_parietal_lobule_L_(A7pc) | -34 | -46 | 50 | 3.29 |
| 38 | Mediovent_occipital_cortex_R_(cCunG) | 8 | -76 | 10 | Fusiform_gyrus_L_(A37lv) | -32 | -64 | -14 | 3.29 |
| 39 | Inferior_parietal_lobule_R_(A39rv) | 58 | -44 | 38 | Superior_parietal_lobule_R_(A7r) | 56 | -40 | 14 | 3.15 |
| 40 | Hippocampus_L_(rHipp) | -26 | -4 | -20 | Lateral_occipital_cortex_L_(OPC) | -46 | -74 | 2 | 3.12 |
| 41 | Amygdala_L_(lAmyg) | -16 | -2 | -20 | Mediovent_occipital_cortex_L_(vmPOS) | -16 | -62 | -8 | 3.05 |

**Table S5**: list of all regions showing significant effects of ongoing symptom count for COVID-19 patients. Brain regions are from the Brainnetome atlas, with centers of mass (CoMs) given in Montréal Neurological Institute (MNI) space coordinates. Number of significant connections is identified, along with the average bootstrap ratio values (BSRs) for these connections.

|  | region | CoM | | | Number of connections | mean BSR |
| --- | --- | --- | --- | --- | --- | --- |
| 1 | Mediovent_occipital_cortex_L_(cCunG) | -4 | -80 | 10 | 11 | 4.17 |
| 2 | Inferior_parietal_lobule_R_(A39c) | 32 | -56 | 52 | 6 | 3.88 |
| 3 | Basal_ganglia_L_(vmPu) | -16 | 4 | -8 | 5 | 3.96 |
| 4 | Orbital_gyrus_R_(A12/47o) | 40 | 38 | -14 | 3 | 4.03 |
| 5 | Inferior_parietal_lobule_R_(A39rv) | 58 | -44 | 38 | 3 | 3.45 |
| 6 | Mediovent_occipital_cortex_L_(vmPOS) | -16 | -62 | -8 | 3 | 3.85 |
| 7 | Superior_temporal_gyrus_L_(A38m) | -32 | 14 | -34 | 2 | 4.03 |
| 8 | Fusiform_gyrus_L_(A37lv) | -32 | -64 | -14 | 2 | 3.97 |
| 9 | Superior_parietal_lobule_R_(A7r) | 56 | -40 | 14 | 2 | 3.38 |
| 10 | Superior_parietal_lobule_L_(A5l) | -16 | -70 | 52 | 2 | 3.80 |
| 11 | Mediovent_occipital_cortex_L_(rCunG) | -10 | -82 | -10 | 2 | 3.96 |
| 12 | Mediovent_occipital_cortex_R_(rCunG) | 10 | -86 | -8 | 2 | 4.08 |
| 13 | Lateral_occipital_cortex_L_(V5/MT+) | -32 | -88 | 10 | 2 | 4.01 |
| 14 | Amygdala_L_(lAmyg) | -16 | -2 | -20 | 2 | 3.70 |
| 15 | Hippocampus_L_(rHipp) | -26 | -4 | -20 | 2 | 3.39 |
| 16 | Superior_frontal_gyrus_R_(A9l) | 14 | 50 | 40 | 1 | 3.52 |
| 17 | Superior_frontal_gyrus_L_(A6dl) | -16 | -2 | 64 | 1 | -4.25 |
| 18 | Orbital_gyrus_R_(A12/47l) | 44 | 32 | -8 | 1 | 4.03 |
| 19 | Precentral_gyrus_L_(A4hf) | -50 | -8 | 40 | 1 | 3.44 |
| 20 | Middle_temporal_gyrus_L_(A37dl) | -58 | -58 | 4 | 1 | 4.07 |
| 21 | Inferior_temporal_gyrus_L_(A20iv) | -44 | -22 | -28 | 1 | 4.78 |
| 22 | Inferior_temporal_gyrus_L_(A37elv) | -50 | -58 | -14 | 1 | 4.10 |
| 23 | Inferior_temporal_gyrus_L_(A37vl) | -56 | -62 | -8 | 1 | 4.03 |
| 24 | Fusiform_gyrus_L_(A20rv) | -56 | -32 | -28 | 1 | 3.88 |
| 25 | Fusiform_gyrus_R_(A37lv) | 32 | -62 | -14 | 1 | 4.05 |
| 26 | Parahippocampal_gyrus_R_(A28/34) | 28 | -28 | -20 | 1 | -4.25 |
| 27 | Superior_parietal_lobule_L_(A7r) | -52 | -50 | 10 | 1 | 3.90 |
| 28 | Superior_parietal_lobule_L_(A7c) | -16 | -58 | 62 | 1 | 3.44 |
| 29 | Superior_parietal_lobule_L_(A7pc) | -34 | -46 | 50 | 1 | 3.29 |
| 30 | Superior_parietal_lobule_L_(A7ip) | -22 | -46 | 64 | 1 | 3.81 |
| 31 | Inferior_parietal_lobule_L_(A39c) | -28 | -58 | 52 | 1 | 3.64 |
| 32 | Inferior_parietal_lobule_L_(A40rd) | -38 | -62 | 46 | 1 | 4.37 |
| 33 | Inferior_parietal_lobule_L_(A40rv) | -46 | -64 | 26 | 1 | 4.05 |
| 34 | Inferior_parietal_lobule_R_(A40rv) | 52 | -56 | 26 | 1 | 3.68 |
| 35 | Postcentral_gyrus_R_(A2) | 56 | -10 | 14 | 1 | 4.08 |
| 36 | Insular_gyrus_R_(dIa) | 32 | 14 | -14 | 1 | 3.51 |
| 37 | Insular_gyrus_R_(dId) | 40 | -8 | 8 | 1 | 4.09 |
| 38 | Cingulate_gyrus_R_(A23c) | 4 | 4 | 38 | 1 | 3.81 |
| 39 | Mediovent_occipital_cortex_R_(cCunG) | 8 | -76 | 10 | 1 | 3.29 |
| 40 | Mediovent_occipital_cortex_R_(rLinG) | 8 | -88 | 14 | 1 | 4.36 |
| 41 | Mediovent_occipital_cortex_R_(vmPOS) | 20 | -62 | -8 | 1 | 3.53 |
| 42 | Lateral_occipital_cortex_L_(OPC) | -46 | -74 | 2 | 1 | 3.12 |
| 43 | Lateral_occipital_cortex_R_(OPC) | 50 | -70 | -2 | 1 | 3.92 |
| 44 | Lateral_occipital_cortex_R_(msOccG) | 32 | -86 | -10 | 1 | 3.64 |
| 45 | Amygdala_R_(lAmyg) | 20 | -2 | -20 | 1 | 4.90 |
| 46 | Hippocampus_R_(rHipp) | 28 | -4 | -20 | 1 | 3.62 |
| 47 | Basal_ganglia_R_(vCa) | 28 | -28 | -10 | 1 | 4.39 |
| 48 | Basal_ganglia_L_(dlPu) | -14 | 2 | 16 | 1 | 3.92 |
