# Supplementary material for: Effects of post-acute COVID-19 syndrome on the functional brain networks of non-hospitalized individuals
Source: Front Neurol. 2023 Mar 27;14:1136408. doi: 10.3389/fneur.2023.1136408 (PMC10083436; doi:10.3389/fneur.2023.1136408)
Supplement: Supplementary file 4 [file Data_Sheet_4.docx]

**Appendix-4: functional connectivity and combined symptoms**

**Methods**

Supplemental analyses were conducted, measuring the bootstrapped partial correlations between functional connectivity and “combined” symptoms (ongoing + resolved) within the COVID-19 group, while adjusting for effects of age and sex. These analyses followed the same approach that was applied to ongoing symptoms, described in Methods section 2.5 of the main text. The use of combined symptoms provides an alternative metric of self-reported post-COVID outcome, which is potentially more reflective of the cumulative effects of SARS-CoV-2 infection over time, as opposed to ongoing symptoms alone.

**Results**

Figure S1 below depicts the associations of combined symptom severity within the COVID-19 group, with 61/29,646 (0.21%) connections showing significant effects at an FDR of 0.05 (see Tables S6, S7 below for details). In Fig. S1(A) the significantly affected connections are shown to consist of mainly positive associations with combined symptoms, with nodes mainly in the medioventral occipital cortex (19 connections total), lateral occipital cortex (12 connections), superior parietal lobule (14 connections), inferior parietal lobule (12 connections), precentral gyrus (8 connections), postcentral gyrus (8 connections) and cingulate cortex (8 connections). The greatest number of positively associated connections are seen in the medioventral occipital cortex (19 connections), superior parietal lobule (14 connections), lateral occipital cortex (12 connections) and inferior parietal lobule (12 connections). Fig S1(B) shows the distribution of connections by lobe, with effects being mainly intra-parietal (p=0.006), occipital-temporal (p<0.001), parietal-occipital (p<0.001), occipital-subcortical (p<0.001), parietal-limbic (p=0.008) and limbic-occipital (p=0.007). Fig. S1(C) plots the mean connectivity values of positive regions against combined symptom count for the COVID-19 group, along with the control group for comparison purposes. The partial correlation coefficient was of moderate strength (ρ=0.59 [0.40, 0.74], p<0.001) with controls showing a similarly positive but weaker effect and relatively wide confidence intervals (ρ=0.20 [-0.49, 0.87], p=0.395).

Comparing the effects of combined symptoms (Figure S1) with ongoing symptoms (Figure 3), similar patterns of positive association are seen between functional connectivity and symptom count, with occipital-temporal, occipital-subcortical and intra-parietal effects being prevalent in both. The increased number of significant connections seen for the combined symptom count analysis suggests that resolved symptoms further contribute to network hyperconnectivity among participants in the COVID-19 group. Furthermore, these effects appear to be primarily supported through enhanced parietal-occipital, parietal-limbic and limbic-occipital connectivity.

**
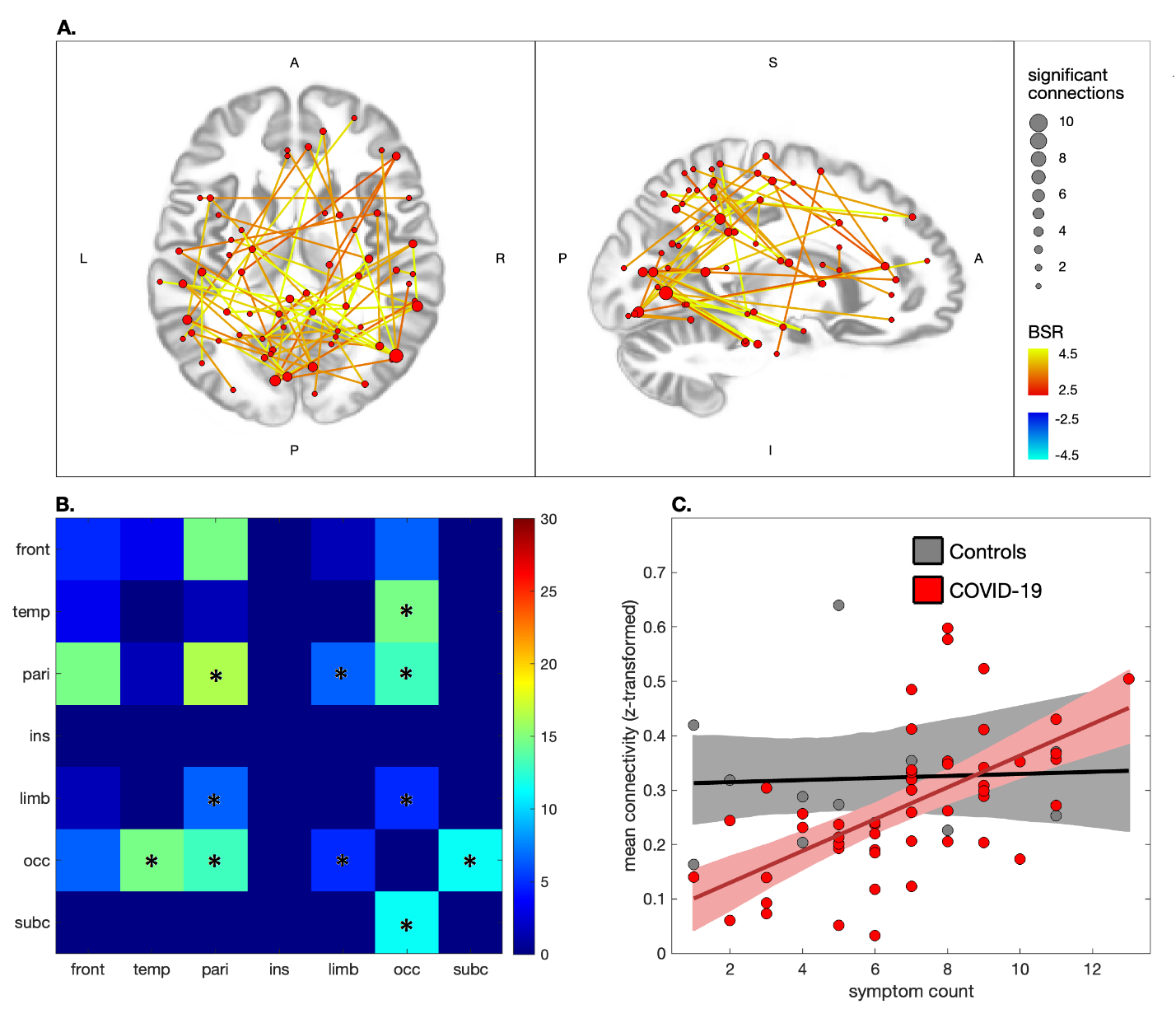
**

**Figure S1**: effects of combined symptom count on functional connectivity for individuals with COVID-19 (ongoing and resolved). (A) significant connections are depicted by lines connecting regions of interest (ROIs), with warm colours indicating increased connectivity with greater symptom scores, and cool colours denoting decreased connectivity; line colours denote the strength of effect in terms of bootstrap ratio (BSR) and node sizes denote the number of significant connections. (B) heatmap showing the percentage of significant connections that are between each pair of lobes; a ‘*’ denotes significantly elevated connections for the lobe pair. (C) scatterplot showing mean connectivity values within regions of significant increase, plotted against symptom count, for individuals in the COVID-19 and control groups; solid lines denote the lines of best fit and shaded areas denote the 95%CIs of the mean.

**Table S6**: pairwise connections showing significant effects of combined symptom count for COVID-19 patients. Brain regions are from the Brainnetome atlas, with centers of mass (CoMs) given in Montréal Neurological Institute (MNI) space coordinates. Bootstrap ratio values (BSRs) provide z-scored standardized measures of effect size.

|  | region 1 | CoM 1 | | | region 2 | CoM 2 | | | BSR |
| --- | --- | --- | --- | --- | --- | --- | --- | --- | --- |
| 1 | Superior_parietal_lobule_L_(A5l) | -16 | -70 | 52 | Superior_frontal_gyrus_R_(A9l) | 14 | 50 | 40 | 5.14 |
| 2 | Hippocampus_R_(rHipp) | 28 | -4 | -20 | Lateral_occipital_cortex_R_(OPC) | 50 | -70 | -2 | 5.07 |
| 3 | Precuneus_L_(A31) | -10 | -68 | 26 | Precentral_gyrus_R_(A4ul) | 34 | -20 | 58 | 4.94 |
| 4 | Mediovent_occipital_cortex_L_(cCunG) | -4 | -80 | 10 | Inferior_temporal_gyrus_L_(A20iv) | -44 | -22 | -28 | 4.91 |
| 5 | Basal_ganglia_R_(vCa) | 28 | -28 | -10 | Mediovent_occipital_cortex_L_(vmPOS) | -16 | -62 | -8 | 4.76 |
| 6 | Postcentral_gyrus_R_(A2) | 56 | -10 | 14 | Inferior_parietal_lobule_R_(A39rv) | 58 | -44 | 38 | 4.76 |
| 7 | Precuneus_R_(A31) | 16 | -64 | 26 | Precentral_gyrus_R_(A4ul) | 34 | -20 | 58 | 4.75 |
| 8 | Basal_ganglia_R_(vCa) | 28 | -28 | -10 | Mediovent_occipital_cortex_R_(vmPOS) | 20 | -62 | -8 | 4.70 |
| 9 | Hippocampus_L_(cHipp) | -22 | -14 | -20 | Lateral_occipital_cortex_R_(OPC) | 50 | -70 | -2 | 4.61 |
| 10 | Superior_parietal_lobule_R_(A7ip) | 22 | -44 | 68 | Middle_temporal_gyrus_L_(A21c) | -64 | -32 | -10 | 4.56 |
| 11 | Lateral_occipital_cortex_R_(OPC) | 50 | -70 | -2 | Inferior_temporal_gyrus_L_(A20iv) | -44 | -22 | -28 | 4.54 |
| 12 | Inferior_parietal_lobule_R_(A39rv) | 58 | -44 | 38 | Superior_parietal_lobule_L_(A7pc) | -34 | -46 | 50 | 4.45 |
| 13 | Lateral_occipital_cortex_L_(OPC) | -46 | -74 | 2 | Inferior_temporal_gyrus_L_(A20iv) | -44 | -22 | -28 | 4.43 |
| 14 | Hippocampus_L_(rHipp) | -26 | -4 | -20 | Mediovent_occipital_cortex_R_(cCunG) | 8 | -76 | 10 | 4.41 |
| 15 | Lateral_occipital_cortex_R_(OPC) | 50 | -70 | -2 | Cingulate_gyrus_L_(A24rv) | -4 | -40 | 32 | 4.33 |
| 16 | Mediovent_occipital_cortex_L_(cCunG) | -4 | -80 | 10 | Superior_parietal_lobule_L_(A7r) | -52 | -50 | 10 | 4.26 |
| 17 | Inferior_temporal_gyrus_L_(A37vl) | -56 | -62 | -8 | Middle_frontal_gyrus_R_(A46) | 28 | 56 | 16 | 4.25 |
| 18 | Mediovent_occipital_cortex_L_(rCunG) | -10 | -82 | -10 | Inferior_parietal_lobule_R_(A39rv) | 58 | -44 | 38 | 4.24 |
| 19 | Inferior_parietal_lobule_R_(A39rv) | 58 | -44 | 38 | Precentral_gyrus_L_(A6cdl) | -32 | -10 | 58 | 4.22 |
| 20 | Precuneus_R_(dmPOS) | 8 | -46 | 58 | Inferior_parietal_lobule_R_(A40rd) | 40 | -64 | 44 | 4.21 |
| 21 | Postcentral_gyrus_R_(A1/2/3tru) | 46 | -26 | 50 | Precuneus_R_(A7m) | 56 | -26 | 26 | 4.17 |
| 22 | Lateral_occipital_cortex_R_(OPC) | 50 | -70 | -2 | Fusiform_gyrus_L_(A20rv) | -56 | -32 | -28 | 4.16 |
| 23 | Superior_parietal_lobule_L_(A7r) | -52 | -50 | 10 | Middle_frontal_gyrus_L_(IFJ) | -40 | 14 | 34 | 4.07 |
| 24 | Cingulate_gyrus_L_(A24rv) | -4 | -40 | 32 | Superior_parietal_lobule_L_(A7ip) | -22 | -46 | 64 | 4.04 |
| 25 | Lateral_occipital_cortex_R_(msOccG) | 32 | -86 | -10 | Cingulate_gyrus_R_(A24rv) | 4 | -38 | 32 | 3.99 |
| 26 | Mediovent_occipital_cortex_R_(cCunG) | 8 | -76 | 10 | Cingulate_gyrus_L_(A24rv) | -4 | -40 | 32 | 3.98 |
| 27 | Mediovent_occipital_cortex_L_(rCunG) | -10 | -82 | -10 | Postcentral_gyrus_L_(A1/2/3ulhf) | -8 | -56 | 34 | 3.95 |
| 28 | Inferior_parietal_lobule_R_(A39rv) | 58 | -44 | 38 | Superior_parietal_lobule_L_(A5l) | -16 | -70 | 52 | 3.93 |
| 29 | Superior_parietal_lobule_L_(A7pc) | -34 | -46 | 50 | Superior_frontal_gyrus_L_(A9m) | -4 | 34 | 38 | 3.90 |
| 30 | Lateral_occipital_cortex_R_(OPC) | 50 | -70 | -2 | Orbital_gyrus_R_(A12/47o) | 40 | 38 | -14 | 3.85 |
| 31 | Inferior_parietal_lobule_L_(A40rd) | -38 | -62 | 46 | Inferior_parietal_lobule_R_(A39c) | 32 | -56 | 52 | 3.85 |
| 32 | Superior_parietal_lobule_R_(A7c) | 20 | -56 | 64 | Middle_frontal_gyrus_L_(IFJ) | -40 | 14 | 34 | 3.85 |
| 33 | Postcentral_gyrus_R_(A2) | 56 | -10 | 14 | Precuneus_L_(dmPOS) | -8 | -46 | 56 | 3.84 |
| 34 | Mediovent_occipital_cortex_R_(cCunG) | 8 | -76 | 10 | Fusiform_gyrus_L_(A20rv) | -56 | -32 | -28 | 3.82 |
| 35 | Postcentral_gyrus_R_(A2) | 56 | -10 | 14 | Precuneus_R_(dmPOS) | 8 | -46 | 58 | 3.78 |
| 36 | Cingulate_gyrus_R_(A23d) | 38 | 4 | 4 | Inferior_parietal_lobule_R_(A40rd) | 40 | -64 | 44 | 3.78 |
| 37 | Mediovent_occipital_cortex_L_(cCunG) | -4 | -80 | 10 | Fusiform_gyrus_L_(A20rv) | -56 | -32 | -28 | 3.77 |
| 38 | Superior_parietal_lobule_R_(A7ip) | 22 | -44 | 68 | Superior_frontal_gyrus_R_(A9l) | 14 | 50 | 40 | 3.76 |
| 39 | Basal_ganglia_R_(dlPu) | 14 | 4 | 14 | Mediovent_occipital_cortex_L_(cLinG) | -4 | 38 | -2 | 3.74 |
| 40 | Lateral_occipital_cortex_L_(msOccG) | -32 | -88 | -14 | Inferior_temporal_gyrus_L_(A37elv) | -50 | -58 | -14 | 3.71 |
| 41 | Mediovent_occipital_cortex_R_(rLinG) | 8 | -88 | 14 | Precentral_gyrus_R_(A4t) | 16 | -22 | 70 | 3.69 |
| 42 | Lateral_occipital_cortex_R_(msOccG) | 32 | -86 | -10 | Inferior_temporal_gyrus_L_(A37elv) | -50 | -58 | -14 | 3.66 |
| 43 | Hippocampus_L_(cHipp) | -22 | -14 | -20 | Mediovent_occipital_cortex_R_(cCunG) | 8 | -76 | 10 | 3.66 |
| 44 | Mediovent_occipital_cortex_R_(cLinG) | 4 | 40 | 4 | Superior_frontal_gyrus_R_(A6dl) | 20 | 4 | 64 | 3.65 |
| 45 | Cingulate_gyrus_L_(A23d) | -38 | 4 | 4 | Precentral_gyrus_R_(A4ul) | 34 | -20 | 58 | 3.58 |
| 46 | Inferior_frontal_gyrus_R_(A44v) | 52 | 14 | 10 | Inferior_frontal_gyrus_L_(A44d) | -46 | 14 | 22 | 3.57 |
| 47 | Postcentral_gyrus_L_(A2) | -56 | -14 | 16 | Precuneus_L_(dmPOS) | -8 | -46 | 56 | 3.55 |
| 48 | Inferior_parietal_lobule_R_(A40rd) | 40 | -64 | 44 | Inferior_parietal_lobule_L_(A39c) | -28 | -58 | 52 | 3.50 |
| 49 | Mediovent_occipital_cortex_L_(rCunG) | -10 | -82 | -10 | Postcentral_gyrus_R_(A1/2/3tru) | 46 | -26 | 50 | 3.50 |
| 50 | Lateral_occipital_cortex_L_(mOccG) | -14 | -68 | 14 | Superior_parietal_lobule_L_(A7r) | -52 | -50 | 10 | 3.48 |
| 51 | Mediovent_occipital_cortex_R_(cLinG) | 4 | 40 | 4 | Superior_parietal_lobule_L_(A7r) | -52 | -50 | 10 | 3.46 |
| 52 | Cingulate_gyrus_R_(A23d) | 38 | 4 | 4 | Postcentral_gyrus_L_(A2) | -56 | -14 | 16 | 3.44 |
| 53 | Mediovent_occipital_cortex_L_(rCunG) | -10 | -82 | -10 | Inferior_parietal_lobule_R_(A39rd) | 46 | -70 | 20 | 3.44 |
| 54 | Precuneus_L_(A31) | -10 | -68 | 26 | Precentral_gyrus_L_(A4ul) | -26 | -26 | 62 | 3.42 |
| 55 | Fusiform_gyrus_L_(A37mv) | -32 | -16 | -32 | Superior_frontal_gyrus_R_(A6dl) | 20 | 4 | 64 | 3.40 |
| 56 | Precentral_gyrus_R_(A4t) | 16 | -22 | 70 | Inferior_frontal_gyrus_R_(IFS) | 46 | 34 | 14 | 3.32 |
| 57 | Mediovent_occipital_cortex_L_(cCunG) | -4 | -80 | 10 | Superior_parietal_lobule_R_(A7r) | 56 | -40 | 14 | 3.31 |
| 58 | Lateral_occipital_cortex_R_(OPC) | 50 | -70 | -2 | Fusiform_gyrus_R_(A20rv) | 52 | -32 | -26 | 3.28 |
| 59 | Mediovent_occipital_cortex_L_(rCunG) | -10 | -82 | -10 | Inferior_frontal_gyrus_R_(IFS) | 46 | 34 | 14 | 3.26 |
| 60 | Cingulate_gyrus_R_(A24rv) | 4 | -38 | 32 | Superior_parietal_lobule_L_(A7c) | -16 | -58 | 62 | 3.25 |
| 61 | Precentral_gyrus_L_(A4ul) | -26 | -26 | 62 | Inferior_frontal_gyrus_R_(IFS) | 46 | 34 | 14 | 3.15 |

**Table S7**: list of all regions showing significant effects of combined symptom count for COVID-19 patients. Brain regions are from the Brainnetome atlas, with centers of mass (CoMs) given in Montréal Neurological Institute (MNI) space coordinates. Number of significant connections is identified, along with the average bootstrap ratio values (BSRs) for these connections.

|  | region | CoM | | | Number of connections | mean BSR |
| --- | --- | --- | --- | --- | --- | --- |
| 1 | Lateral_occipital_cortex_R_(OPC) | 50 | -70 | -2 | 7 | 4.26 |
| 2 | Inferior_parietal_lobule_R_(A39rv) | 58 | -44 | 38 | 5 | 4.32 |
| 3 | Mediovent_occipital_cortex_L_(rCunG) | -10 | -82 | -10 | 5 | 3.68 |
| 4 | Superior_parietal_lobule_L_(A7r) | -52 | -50 | 10 | 4 | 3.82 |
| 5 | Mediovent_occipital_cortex_L_(cCunG) | -4 | -80 | 10 | 4 | 4.06 |
| 6 | Mediovent_occipital_cortex_R_(cCunG) | 8 | -76 | 10 | 4 | 3.97 |
| 7 | Inferior_frontal_gyrus_R_(IFS) | 46 | 34 | 14 | 3 | 3.25 |
| 8 | Precentral_gyrus_R_(A4ul) | 34 | -20 | 58 | 3 | 4.42 |
| 9 | Inferior_temporal_gyrus_L_(A20iv) | -44 | -22 | -28 | 3 | 4.63 |
| 10 | Fusiform_gyrus_L_(A20rv) | -56 | -32 | -28 | 3 | 3.92 |
| 11 | Inferior_parietal_lobule_R_(A40rd) | 40 | -64 | 44 | 3 | 3.83 |
| 12 | Postcentral_gyrus_R_(A2) | 56 | -10 | 14 | 3 | 4.13 |
| 13 | Cingulate_gyrus_L_(A24rv) | -4 | -40 | 32 | 3 | 4.11 |
| 14 | Superior_frontal_gyrus_R_(A9l) | 14 | 50 | 40 | 2 | 4.45 |
| 15 | Superior_frontal_gyrus_R_(A6dl) | 20 | 4 | 64 | 2 | 3.52 |
| 16 | Middle_frontal_gyrus_L_(IFJ) | -40 | 14 | 34 | 2 | 3.96 |
| 17 | Precentral_gyrus_L_(A4ul) | -26 | -26 | 62 | 2 | 3.29 |
| 18 | Precentral_gyrus_R_(A4t) | 16 | -22 | 70 | 2 | 3.51 |
| 19 | Inferior_temporal_gyrus_L_(A37elv) | -50 | -58 | -14 | 2 | 3.69 |
| 20 | Superior_parietal_lobule_L_(A5l) | -16 | -70 | 52 | 2 | 4.53 |
| 21 | Superior_parietal_lobule_L_(A7pc) | -34 | -46 | 50 | 2 | 4.17 |
| 22 | Superior_parietal_lobule_R_(A7ip) | 22 | -44 | 68 | 2 | 4.16 |
| 23 | Precuneus_L_(dmPOS) | -8 | -46 | 56 | 2 | 3.70 |
| 24 | Precuneus_R_(dmPOS) | 8 | -46 | 58 | 2 | 4.00 |
| 25 | Precuneus_L_(A31) | -10 | -68 | 26 | 2 | 4.18 |
| 26 | Postcentral_gyrus_L_(A2) | -56 | -14 | 16 | 2 | 3.49 |
| 27 | Postcentral_gyrus_R_(A1/2/3tru) | 46 | -26 | 50 | 2 | 3.84 |
| 28 | Cingulate_gyrus_R_(A23d) | 38 | 4 | 4 | 2 | 3.61 |
| 29 | Cingulate_gyrus_R_(A24rv) | 4 | -38 | 32 | 2 | 3.62 |
| 30 | Mediovent_occipital_cortex_R_(cLinG) | 4 | 40 | 4 | 2 | 3.56 |
| 31 | Lateral_occipital_cortex_R_(msOccG) | 32 | -86 | -10 | 2 | 3.83 |
| 32 | Hippocampus_L_(cHipp) | -22 | -14 | -20 | 2 | 4.13 |
| 33 | Basal_ganglia_R_(vCa) | 28 | -28 | -10 | 2 | 4.73 |
| 34 | Superior_frontal_gyrus_L_(A9m) | -4 | 34 | 38 | 1 | 3.90 |
| 35 | Middle_frontal_gyrus_R_(A46) | 28 | 56 | 16 | 1 | 4.25 |
| 36 | Inferior_frontal_gyrus_L_(A44d) | -46 | 14 | 22 | 1 | 3.57 |
| 37 | Inferior_frontal_gyrus_R_(A44v) | 52 | 14 | 10 | 1 | 3.57 |
| 38 | Orbital_gyrus_R_(A12/47o) | 40 | 38 | -14 | 1 | 3.85 |
| 39 | Precentral_gyrus_L_(A6cdl) | -32 | -10 | 58 | 1 | 4.22 |
| 40 | Middle_temporal_gyrus_L_(A21c) | -64 | -32 | -10 | 1 | 4.56 |
| 41 | Inferior_temporal_gyrus_L_(A37vl) | -56 | -62 | -8 | 1 | 4.25 |
| 42 | Fusiform_gyrus_R_(A20rv) | 52 | -32 | -26 | 1 | 3.28 |
| 43 | Fusiform_gyrus_L_(A37mv) | -32 | -16 | -32 | 1 | 3.40 |
| 44 | Superior_parietal_lobule_R_(A7r) | 56 | -40 | 14 | 1 | 3.31 |
| 45 | Superior_parietal_lobule_L_(A7c) | -16 | -58 | 62 | 1 | 3.25 |
| 46 | Superior_parietal_lobule_R_(A7c) | 20 | -56 | 64 | 1 | 3.85 |
| 47 | Superior_parietal_lobule_L_(A7ip) | -22 | -46 | 64 | 1 | 4.04 |
| 48 | Inferior_parietal_lobule_L_(A39c) | -28 | -58 | 52 | 1 | 3.50 |
| 49 | Inferior_parietal_lobule_R_(A39c) | 32 | -56 | 52 | 1 | 3.85 |
| 50 | Inferior_parietal_lobule_R_(A39rd) | 46 | -70 | 20 | 1 | 3.44 |
| 51 | Inferior_parietal_lobule_L_(A40rd) | -38 | -62 | 46 | 1 | 3.85 |
| 52 | Precuneus_R_(A7m) | 56 | -26 | 26 | 1 | 4.17 |
| 53 | Precuneus_R_(A31) | 16 | -64 | 26 | 1 | 4.75 |
| 54 | Postcentral_gyrus_L_(A1/2/3ulhf) | -8 | -56 | 34 | 1 | 3.95 |
| 55 | Cingulate_gyrus_L_(A23d) | -38 | 4 | 4 | 1 | 3.58 |
| 56 | Mediovent_occipital_cortex_L_(cLinG) | -4 | 38 | -2 | 1 | 3.74 |
| 57 | Mediovent_occipital_cortex_R_(rLinG) | 8 | -88 | 14 | 1 | 3.69 |
| 58 | Mediovent_occipital_cortex_L_(vmPOS) | -16 | -62 | -8 | 1 | 4.76 |
| 59 | Mediovent_occipital_cortex_R_(vmPOS) | 20 | -62 | -8 | 1 | 4.70 |
| 60 | Lateral_occipital_cortex_L_(mOccG) | -14 | -68 | 14 | 1 | 3.48 |
| 61 | Lateral_occipital_cortex_L_(OPC) | -46 | -74 | 2 | 1 | 4.43 |
| 62 | Lateral_occipital_cortex_L_(msOccG) | -32 | -88 | -14 | 1 | 3.71 |
| 63 | Hippocampus_L_(rHipp) | -26 | -4 | -20 | 1 | 4.41 |
| 64 | Hippocampus_R_(rHipp) | 28 | -4 | -20 | 1 | 5.07 |
| 65 | Basal_ganglia_R_(dlPu) | 14 | 4 | 14 | 1 | 3.74 |
